# Plasma and Serum Metagenomic Sequencing of Microbial Cell-Free Nucleic Acids for Suspected Central Nervous System Infection: A Systematic Review

**DOI:** 10.3390/diagnostics16203375

**Published:** 2026-10-16

**Authors:** Felix Bratosin, Horia Silviu Branea, Elena Hogea

**Affiliations:** 1 Division of Infectious Diseases, Department of Internal Medicine, McGovern Medical School, The University of Texas Health Science Center at Houston (UTHealth Houston), Houston, TX 77030, USA; felix.bratosin@uth.tmc.edu; 2 Department of Internal Medicine I-Medical Semiology II, Faculty of Medicine, “Victor Babeș” University of Medicine and Pharmacy Timișoara, 2 Eftimie Murgu Square, 300041 Timișoara, Romania; 3 Department of Microbiology, Faculty of Medicine, “Victor Babeș” University of Medicine and Pharmacy Timișoara, 2 Eftimie Murgu Square, 300041 Timișoara, Romania; hogea.elena@umft.ro

**Keywords:** cell-free nucleic acids, metagenomics, central nervous system infections, meningitis, encephalitis, brain abscess, high-throughput nucleotide sequencing, diagnostic stewardship

## Abstract

**Background/Objectives:** Untargeted metagenomic sequencing of microbial cell-free DNA or RNA in plasma or serum offers pathogen detection without lumbar puncture or biopsy, but its value in suspected central nervous system (CNS) infection is uncertain. We reviewed diagnostic yield, agreement with CNS-site testing, clinical impact, and failure modes. **Methods:** PubMed/MEDLINE was searched with seven documented strings (inception to 2 September 2026), supplemented by citation and web searching. Eligible reports were primary human studies of untargeted plasma or serum metagenomic sequencing with a separable result in patients with suspected or confirmed infectious CNS syndromes. Selection, extraction, and design-specific risk-of-bias appraisal followed PRISMA 2020 and were performed independently by two authors with disagreements resolved by discussion; the search was restricted to PubMed/MEDLINE and no protocol was registered. Synthesis was narrative because designs and reference standards were incompatible. **Results:** Of 190 reports assessed, 69 studies were included (18 denominator-based cohorts, 8 small series, 43 case reports; 51 from the United States). Positive CNS-specific clinical impact in commercial assay cohorts ranged from 0 of 24 adult to 5 of 24 pediatric meningoencephalitis tests. Among 51 case-level reports, the plasma or serum result was concordant with CNS-site testing in 28, positive when CNS-site testing was negative in 5, unconfirmed in 12, and negative or missed the pathogen in 6. All five plasma-only detections came from single case reports, which contributed to no yield or agreement denominator; excluding the four abstract-level case reports moved no proportion by more than 1.3 percentage points. RNA viruses, low-burden focal mould disease, and chromosomally integrated HHV-6 were recurrent sources of false-negative or misleading results. **Conclusions:** Plasma or serum metagenomic sequencing is a useful adjunct in selected difficult CNS infections, particularly disseminated, fungal, amoebic, or fastidious bacterial disease, but it does not replace CNS-site diagnostics.

## 1. Introduction

Infections of the central nervous system (CNS) remain a major cause of death and disability worldwide: meningitis caused an estimated 318,400 deaths in 2016, and in a large United States hospital database more than 26,000 adults were admitted with meningitis or encephalitis over four years, with no etiology identified in one-fifth [1,2]. Outcomes depend on the speed with which pathogen-directed therapy is started, which is why contemporary guidelines for bacterial meningitis and encephalitis emphasize rapid cerebrospinal fluid (CSF) analysis and empirical treatment before confirmation [3,4,5]. Despite structured diagnostic algorithms and multiplex CSF polymerase chain reaction (PCR) panels, an etiology is not identified in 37% to 63% of encephalitis cases and in a substantial fraction of aseptic meningitis and brain abscess presentations [6,7,8,9,10,11]. Prior antimicrobial exposure, low organism burden, unexpected or fastidious pathogens, compartmentalized focal disease, and contraindications to lumbar puncture or biopsy each contribute to this diagnostic gap. The gap is not uniform by age. Neonates and infants present non-specifically, yield small cerebrospinal fluid volumes, and are often sampled after antibiotics, but have concurrent bacteraemia far more often than older patients; in adults, the gap is driven instead by prior therapy, immunosuppression, focal parenchymal disease in which the subarachnoid space may be uninvolved, and contraindications to lumbar puncture.

Untargeted metagenomic next-generation sequencing (mNGS) addresses the hypothesis-free detection problem by sequencing all nucleic acid in a specimen and computationally identifying microbial reads [12,13]. In CSF, clinically validated mNGS increases etiologic yield in meningitis and encephalitis, but it still requires lumbar puncture and samples only the subarachnoid compartment [14,15,16]. Sequencing of microbial cell-free DNA (mcfDNA) in plasma exploits the release of short microbial DNA fragments into the circulation from infected tissue; a commercial assay has been analytically validated and is now used across a broad range of infectious syndromes, with tens of thousands of clinical tests reported [17,18,19]. Multicenter clinical-impact studies in unselected patients with suspected infection show that positive results change management in a minority of cases, and expert recommendations increasingly stress diagnostic stewardship and careful interpretation of low-level, commensal, or latent-virus signals [20,21,22]. Targeted blood amplification was not treated as an index test or comparator here: it requires an a priori organism hypothesis, reports on a scale that cannot be combined with untargeted read-based output, and in CNS disease its targets largely duplicate those of multiplex cerebrospinal fluid panels.

Shedding into blood differs by compartment. A pyogenic abscess releases organisms mainly during early cerebritis and the antecedent bacteraemia, because the maturing capsule progressively isolates the purulent core from the subarachnoid space and the circulation [11]. Ventriculitis sustains a large microbial burden in continuity with flowing cerebrospinal fluid and the choroid plexus, giving more direct access to blood but also making cerebrospinal fluid the higher-yield specimen [23]; in disseminated angioinvasive infection, blood is a primary rather than a surrogate compartment. Whether these general findings transfer to the CNS is unclear. Plasma testing might detect organisms that shed into blood from brain abscesses, vasculitic lesions, or disseminated fungal and amoebic infection when CSF is nondiagnostic, yet the blood–brain barrier could equally prevent detection of localized low-burden disease. Barrier integrity favours detection during the acute phase: in adults with community-acquired bacterial meningitis, the cerebrospinal fluid to serum albumin quotient is markedly raised, with reported means of 22.8 ± 16.2 and 44.4 ± 39.5 in patients with favourable and unfavourable outcomes against upper limits of normal of about 6.5 to 8 × 10^−3^ [24]. Microbial cell-free DNA fragments are far smaller than albumin, so a barrier open to serum protein should also release microbial nucleic acid into plasma, and the converse predicts low yield once inflammation resolves or disease is encapsulated. Existing syntheses do not resolve this question: a meta-analysis of plasma cell-free mNGS pooled heterogeneous syndromes without CNS-specific estimates [25], and reviews of metagenomics in meningitis and encephalitis have concentrated on CSF applications [26,27]. No systematic review has been dedicated to plasma or serum metagenomic sequencing in suspected CNS infection.

The primary objective of this systematic review was to determine, in children and adults evaluated for meningitis, encephalitis, ventriculitis, intracranial or spinal epidural abscess, infection-related CNS vasculitis, or other neuroinvasive infection, how often untargeted plasma or serum metagenomic sequencing identifies a plausible causative pathogen and how its result relates to CNS-site reference testing. The comparator was CNS-site reference testing as performed in practice: culture of cerebrospinal fluid, brain tissue, or abscess material together with targeted cerebrospinal fluid amplification constituted the primary reference standard, cerebrospinal fluid or tissue metagenomics counted as a reference only where the source study used it as the diagnostic test of record, and clinical adjudication alone was the lowest tier. Secondary objectives were to describe turnaround time, documented effects on management, and the circumstances of false-negative, discordant, or incidental results, and to establish whether the published data are homogeneous enough to justify quantitative pooling. We hypothesized that yield and impact would be concentrated in disseminated or tissue-invasive infections and in patients in whom CNS-site sampling had failed, rather than in unselected meningoencephalitis.

## 2. Materials and Methods

### 2.1. Design and Reporting

This systematic review followed the PRISMA 2020 statement [28]. The completed PRISMA 2020 checklist is provided in Table S1 (Supplementary Materials). It was not registered prospectively in PROSPERO or an equivalent registry, a departure from PRISMA 2020 item 24a; the protocol, search exports, screening decisions, extraction, and appraisal are retained in an audit workbook that accompanies the manuscript. The question was framed with the PICOS structure. Population: humans of any age with suspected or confirmed infectious CNS syndrome (meningitis including aseptic meningitis, meningoencephalitis, encephalitis, rhombencephalitis, ventriculitis, brain, intracranial, or spinal epidural abscess, infection-related CNS vasculitis or mycotic aneurysm, or other neuroinvasive infection). Index test: untargeted or broad-range metagenomic sequencing of cell-free nucleic acid from plasma or serum, whether commercial or laboratory-developed. Comparator: any CNS-site or conventional reference (culture, targeted PCR, CSF or tissue metagenomics, histopathology, clinical adjudication), or none. Outcomes: organism detected, agreement with the reference, diagnostic yield, turnaround time, management change, and patient outcome. Study designs: cohort, cross-sectional, case–control, case series, and case reports. The primary reference standard was prespecified as culture of a CNS specimen or targeted cerebrospinal fluid amplification. Studies whose only comparator was clinical adjudication were retained descriptively but recorded as positive without CNS-site confirmation rather than as agreement, excluded from concordance statements, and rated high concern in the reference-standard domain; adjudication that incorporated the index test was recorded as incorporation bias. Case reports and single-patient series were excluded from every calculation of yield or agreement and used only to describe pathogens, failure modes, and management consequences.

### 2.2. Eligibility Criteria

Reports were included when all of the following were met: (i) primary human clinical report; (ii) the index test was untargeted metagenomic sequencing performed on plasma or serum, stated explicitly (a named commercial plasma mcfDNA assay qualified); (iii) at least one patient had an eligible CNS syndrome; (iv) the plasma or serum result for that patient or subgroup was reported and separable from non-CNS patients; and (v) the full text was accessible, or, when it was not, the published abstract itself reported the plasma or serum result for a CNS patient, in which case the report was included as an abstract-level extraction and flagged. Reports were excluded when sequencing was performed only on CSF, tissue, or whole-blood cellular DNA, or was targeted (PCR, 16S, probe-capture panels); when the blood fraction was described only as “blood” without specifying plasma, serum, or cell-free nucleic acid; when no CNS syndrome was present, including endocarditis with embolic stroke but no documented CNS infection; when CNS patients existed but no separable plasma or serum result was reported; or when the record was a review, editorial, letter without primary data, conference abstract, protocol, or non-human study. No language, date, or country restriction was applied. Reports describing the same patients were counted once.

### 2.3. Information Sources and Search Strategy

PubMed/MEDLINE was searched on 2 September 2026 from database inception using seven strings that combined specimen terms (plasma, blood, serum), technology terms (cell-free DNA, cfDNA, mcfDNA, Karius, metagenomic next-generation sequencing, mNGS, next-generation sequencing), and CNS syndrome terms (meningitis, encephalitis, meningoencephalitis, brain or intracranial abscess, ventriculitis, central nervous system, neuroinvasive), with additional strings capturing general infection cohorts of plasma cell-free sequencing and case reports; the complete strings and record counts are reproduced in Table A1. Embase, Scopus, and Web of Science required subscriptions that were unavailable. The Cochrane Library and Europe PMC are freely searchable, so their omission reflects reviewer capacity rather than access; preprints, theses, and non-MEDLINE journals may therefore have been missed, which compounds rather than offsets the exclusion of reports specifying only blood and biases the included set toward United States commercial testing. Google Scholar was not used. Web searching with the general Google search engine paired assay names with syndrome names, but was exploratory and was not logged to the standard that PRISMA 2020 item 7 requires, so query dates and the number of result pages inspected are not reported and this route is not reproducible as specified; its yield is identifiable instead as the reports entering through the other-methods arm. Supplementary searching further comprised backward screening of the reference lists of the included reports and of prior reviews [25,26,27], and forward screening of reports citing early plasma-sequencing CNS cases. Records identified only through these routes are reported in the “other methods” arm of the flow diagram.

### 2.4. Selection Process

Records were deduplicated by PubMed identifier with a script. Titles and abstracts were screened against the PICOS criteria with a deliberately inclusive rule: any report of plasma or blood metagenomic sequencing with a possible CNS syndrome or subgroup proceeded to full-text assessment. Full texts were obtained from PubMed Central or publisher sites; each was assessed against criteria (i)–(v) and one primary exclusion reason was recorded. Title-and-abstract screening, full-text eligibility assessment, data extraction, and risk-of-bias appraisal were each performed independently by two authors, who assessed all records separately and then compared decisions; disagreements were resolved by discussion against the source. No formal inter-rater agreement statistic was calculated. Exactly one primary exclusion reason was recorded for every report excluded or sought and not retrieved. Overlap was checked across all included reports using explicit statements, shared authorship, shared institution, and overlapping dates in combination, and every pair sharing an author or institution was then compared on age, sex, syndrome, organism, and presentation date. Overlapping data were not double counted. The method cannot exclude undeclared overlap between reports sharing neither author nor institution.

### 2.5. Data Extraction and Risk of Bias

For every included report, the following variables were extracted verbatim where possible: country, design, study period, total and CNS-specific denominators, age group, immune status, CNS syndrome, specimen wording, assay, reference standard, organisms detected in plasma or serum, numerical yield or agreement, turnaround time, management effect, follow-up and outcome, overlap, and source of extraction (full text or abstract). Values not reported were coded NR and were never imputed. Denominator-based cohorts were appraised with QUADAS-2 domains (patient selection, index test, reference standard, flow and timing) supplemented by whether clinical-impact criteria were prespecified and adjudicated independently of the index test [29]. Because QUADAS-2 yields low, high, or unclear judgements rather than a graded score, each study was also assigned a three-category overall judgement by a prespecified mapping: low and low–moderate to low, moderate and above to high, and unclear where a domain could not be judged. The three-category judgement is the QUADAS-2 result; the descriptive labels are retained only for the reason assigned and are not a validated score. Case reports and small series were appraised with the domains proposed for case-level evidence: selection, ascertainment of exposure and outcome, causality (including independent confirmation of the organism at the CNS site), and reporting [30]. An overall qualitative concern (low, low–moderate, moderate, moderate–high, high) was assigned with a stated reason; abstract-level extractions were rated high. Quantitative output could not be placed on a common scale: studies reported molecules per microlitre, reads per million, raw counts, cycle thresholds, or nothing at all; no raw sequence data were available, and no standardized cross-platform threshold for positivity exists. Every quantitative result was therefore recoded as positive, negative, or inconclusive, where positive denotes a detection at or above the study’s own reporting threshold and attributed to the syndrome, negative no organism above threshold, and inconclusive a subthreshold detection, one the study declined to attribute, or any result reported without a stated threshold. Original values are retained but are nowhere compared across studies.

### 2.6. Synthesis

Meta-analysis was prespecified as appropriate only if at least two studies shared a compatible population, index-test definition, independent reference standard, and extractable 2 × 2 data. This condition was not met, and no summary sensitivity, specificity, or certainty rating was calculated. Synthesis was therefore structured and narrative, stratified into denominator-based cohorts, small series with per-patient data, and single case reports. For case-level reports, the relationship of the plasma or serum result to CNS-site testing was categorized as concordant with CSF or tissue, positive with negative CNS-site testing, positive without CNS-site confirmation, or negative or missed pathogen. Study-level proportions are presented with their original numerators and denominators; no proportion was pooled across studies. Heterogeneity was handled descriptively, and prespecified subgroups were adults versus children, immunocompromised hosts, diffuse meningeal or encephalitic versus focal disease, and commercial versus research assays. Denominator-based studies were subclassified as unselected or positively selected, and only unselected denominators support statements about yield or agreement. Relations to CNS-site testing were further classified by the compartment in which confirmation occurred, recorded as cerebrospinal fluid, brain or meningeal tissue, both, or not specified, and by whether the plasma result preceded the reference test.

## 3. Results

### 3.1. Study Selection

**Figure 1:**
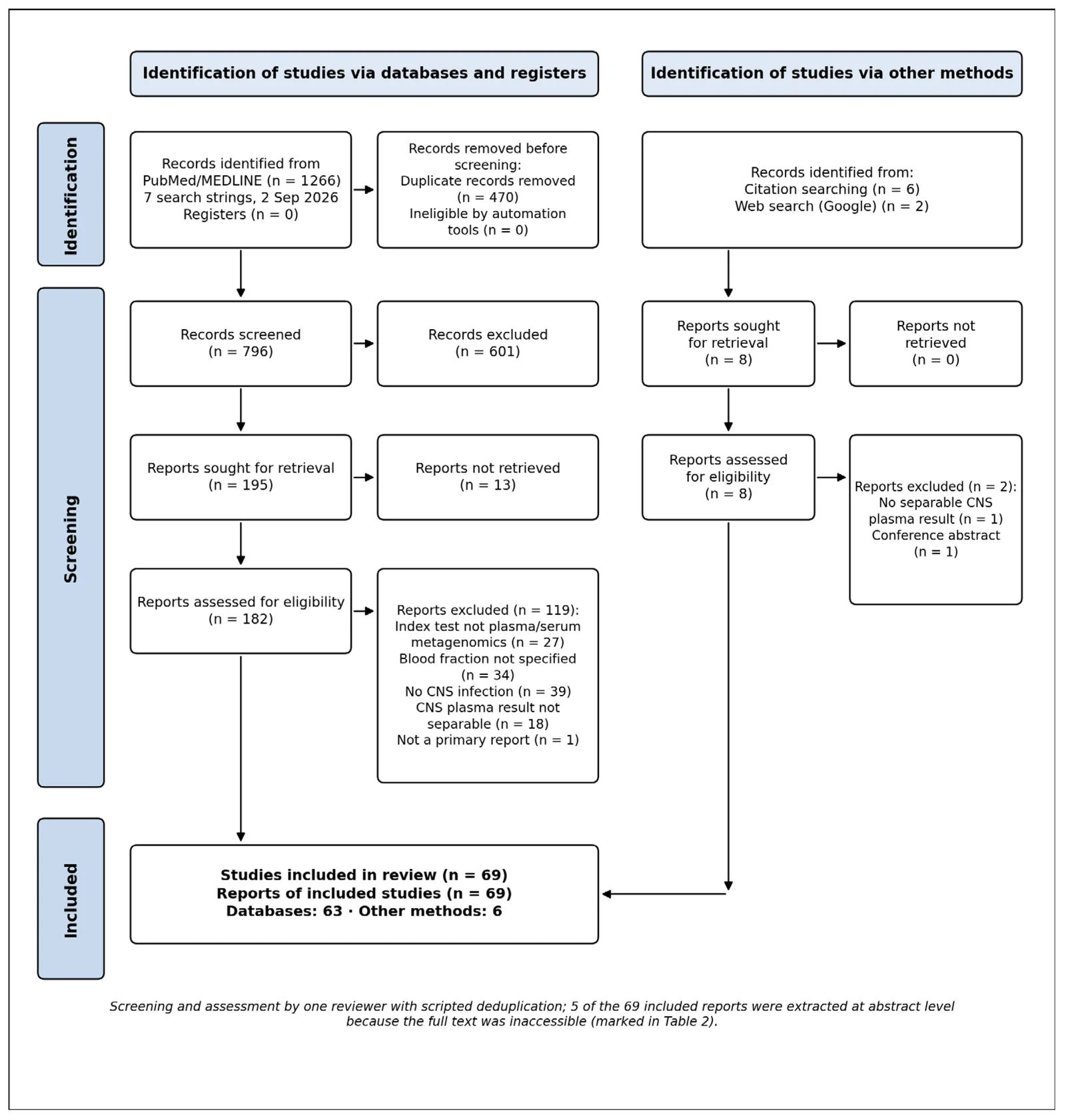
PRISMA 2020 flow diagram.

The seven PubMed strings retrieved 1266 records, of which 796 were unique. After title and abstract screening, 195 reports were sought and 182 were assessed at full text or, for five reports with inaccessible full text, at abstract level; 13 reports could not be retrieved. Eight further reports were identified by citation and web searching. In total, 69 studies were included, and 121 reports were excluded after assessment, most often because no CNS syndrome was present, because the blood fraction was not specified, or because sequencing was not performed on plasma or serum (Figure 1). The excluded reports and their primary reasons are listed in Table A2. Of the 182 reports assessed through the database arm, 119 were excluded and 63 included; of the 8 reports identified by citation and web searching, 2 were excluded and 6 included, giving 121 exclusions and 69 inclusions from 190 assessed reports. The 13 reports that could not be retrieved all carry PubMed identifiers and were published between 2020 and 2026, and were sought through PubMed Central and publisher sites only, so they are reported as not retrieved by the routes used rather than as inaccessible.

### 3.2. Characteristics of Included Studies

Table 1 summarizes the 69 included studies: 18 denominator-based cohorts or series, 8 small series with per-patient data, and 43 single case reports, published between 2016 and 2026. Fifty-one originated in the United States and 13 in China, with single reports from Poland, Japan, the United Kingdom, Switzerland, and Germany. CNS-specific denominators in the cohorts were small, ranging from 1 to 78 tests within cohorts of 30 to 1000 tests, and the largest dedicated CNS denominators came from research studies of paired blood and CSF sequencing in children with meningitis or encephalitis. Populations included immunocompromised transplant and oncology patients, neonates and infants, and immunocompetent hosts with rare pathogens. Syndromes spanned bacterial, viral, fungal, mycobacterial, parasitic, and free-living amoebic meningitis, encephalitis, brain abscess, ventriculitis, infectious intracranial aneurysm or vasculitis, and spinal epidural infection. Age was recoded into the four standard categories, neonates 0 to 28 days, infants 29 days to under 1 year, children 1 to under 18 years, and adults 18 years and over, with adolescents and toddlers mapped to children and the verbatim value retained alongside; one cohort reported only children aged 0 to 14 years, a range that also spans the younger categories. Immune status was recoded as immunocompetent, immunocompromised, mixed, or not reported, and for cohorts reporting only a percentage, the corresponding count is given, derived from that percentage and the cohort denominator and marked as derived rather than extracted.

### 3.3. Index Tests and Reference Standards

Table 2 lists the specimen, assay, reference standard, and data source for each study. The commercial plasma mcfDNA assay (Karius Test; Karius, Inc., Redwood City, CA, USA) was named explicitly in 41 studies, with 2 further reports describing a plasma microbial cell-free DNA assay reporting in molecules per microlitre without naming a vendor, so 43 is the maximum consistent with the published descriptions; the remainder used laboratory-developed DNA or RNA metagenomic workflows on plasma or serum, including research pipelines in outbreak investigations and paired specimen studies. Serum was the specimen in 11 studies. Reference standards ranged from CSF culture and multiplex PCR to CSF or tissue metagenomics, histopathology, public-health laboratory PCR, and clinical adjudication, and were not interchangeable. Five studies were extracted at abstract level.

### 3.4. Findings in Denominator-Based Cohorts

**Figure 2:**
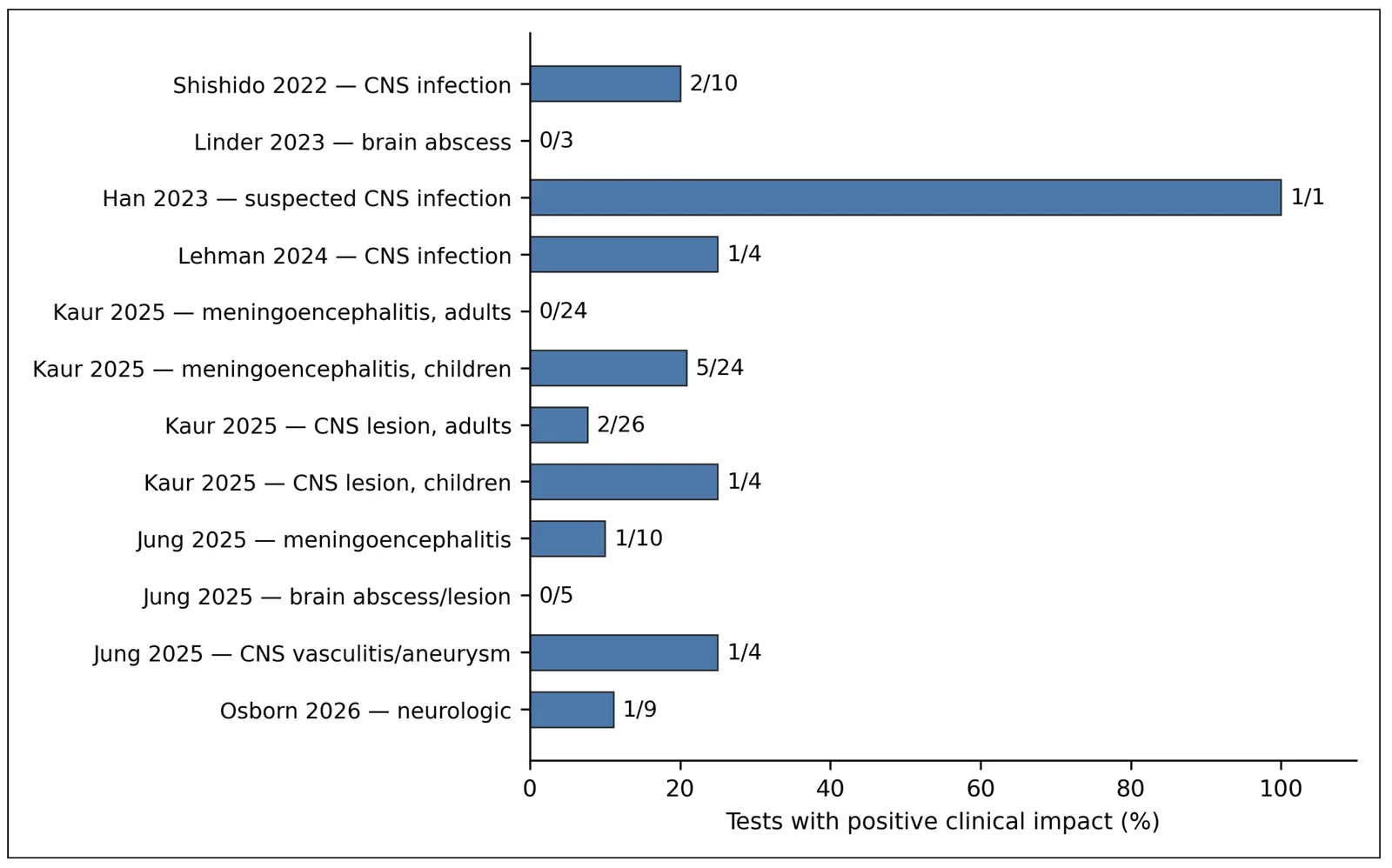
Positive clinical impact in CNS subgroups. Data from Shishido 2022 [34], Han 2023 [36], Linder 2023 [37], Lehman 2024 [40], Kaur 2025 [42], Jung 2025 [43], and Osborn 2026 [46].

All proportions in this section derive from the 18 denominator-based cohorts; no case report and no positively selected series contribute to any denominator. CNS-specific findings of the 18 cohorts are reported in Table 3. In the only cohort restricted to meningitis and tested with a commercial assay, plasma sequencing identified a causative pathogen in 36% of 28 children, conventional testing in 50%, and the combination in 57%. Positive clinical impact in CNS subgroups of clinical-impact cohorts is shown in Figure 2: 0 of 24 adult and 5 of 24 pediatric meningoencephalitis tests, 2 of 26 adult and 1 of 4 pediatric CNS-lesion tests, 2 of 10 meningoencephalitis tests in one centre, and 1 of 10 meningoencephalitis, 0 of 5 brain-abscess, and 1 of 4 CNS-vasculitis tests in another; 0 of 3 brain-abscess tests, 1 of 4 and 1 of 9 CNS-indication tests, and 1 of 1 suspected CNS infection in smaller subgroups. Two conventionally confirmed pediatric CNS infections (tuberculous meningitis, HSV-1) were missed by plasma sequencing in one cohort, and 0 of 3 Powassan encephalitis cases were detected by a DNA-only assay. In paired specimen studies, blood cell-free sequencing was positive in 19 of 27 neonates with CNS–bloodstream coinfection versus 20 of 27 for CSF, serum virome sequencing detected a viral pathogen in 3 of 113 children versus 9 of 113 for CSF with no paired concordance, plasma sequencing of children with bacterial meningitis reproduced the CSF dominant species in 9 of 9 *Klebsiella*-predominant and 9 of 17 other cases, and serum or plasma sequencing added no pathogen beyond CSF in 20 children with encephalitis. Plasma sequencing was positive in 4 of 10 meningitis patients in a multicenter Chinese cohort and in all CNS cases of two series selected on positive results.

### 3.5. Findings in Case-Level Reports

**Figure 3:**
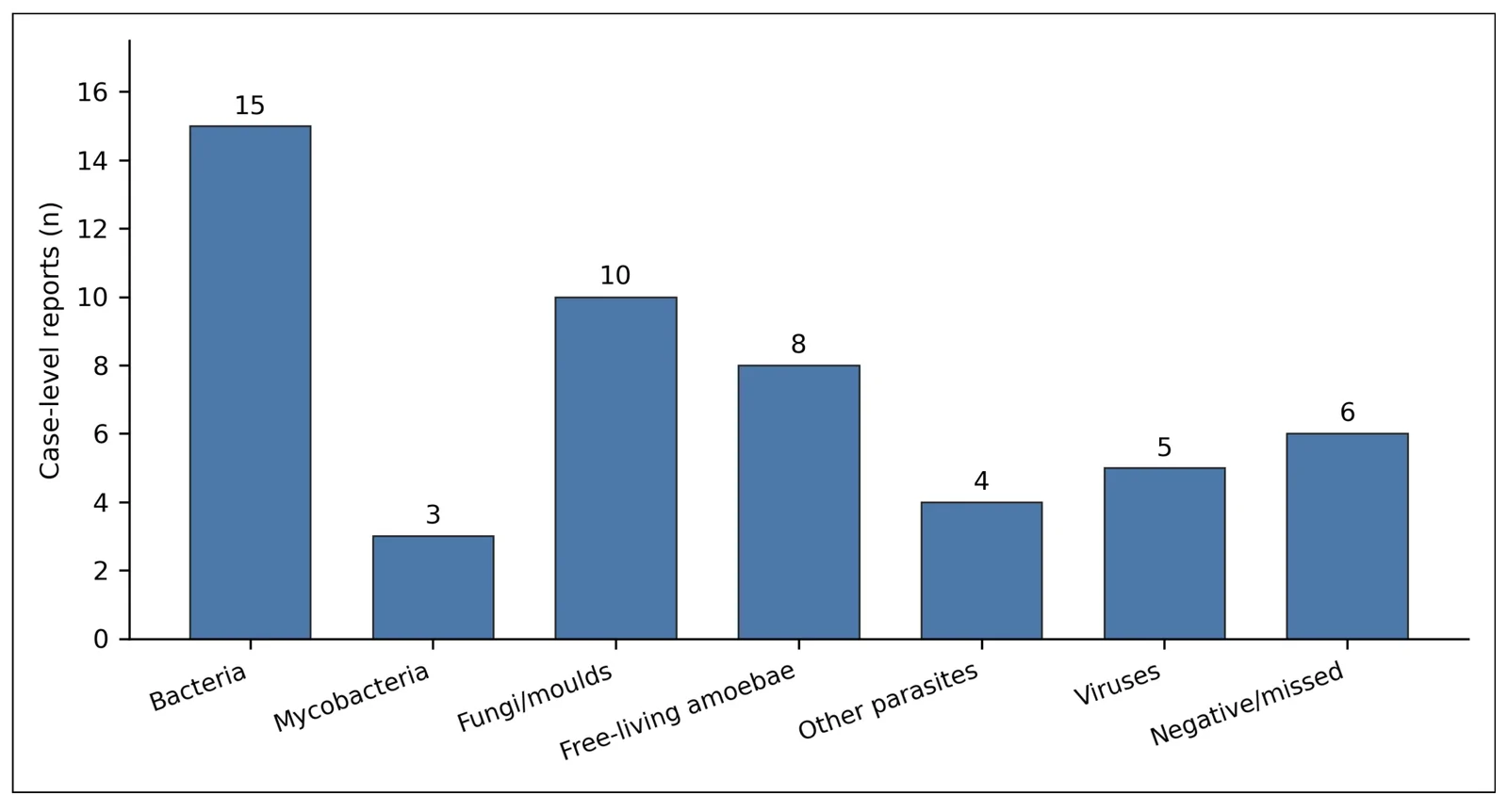
Principal organism class detected in plasma or serum.

**Figure 4:**
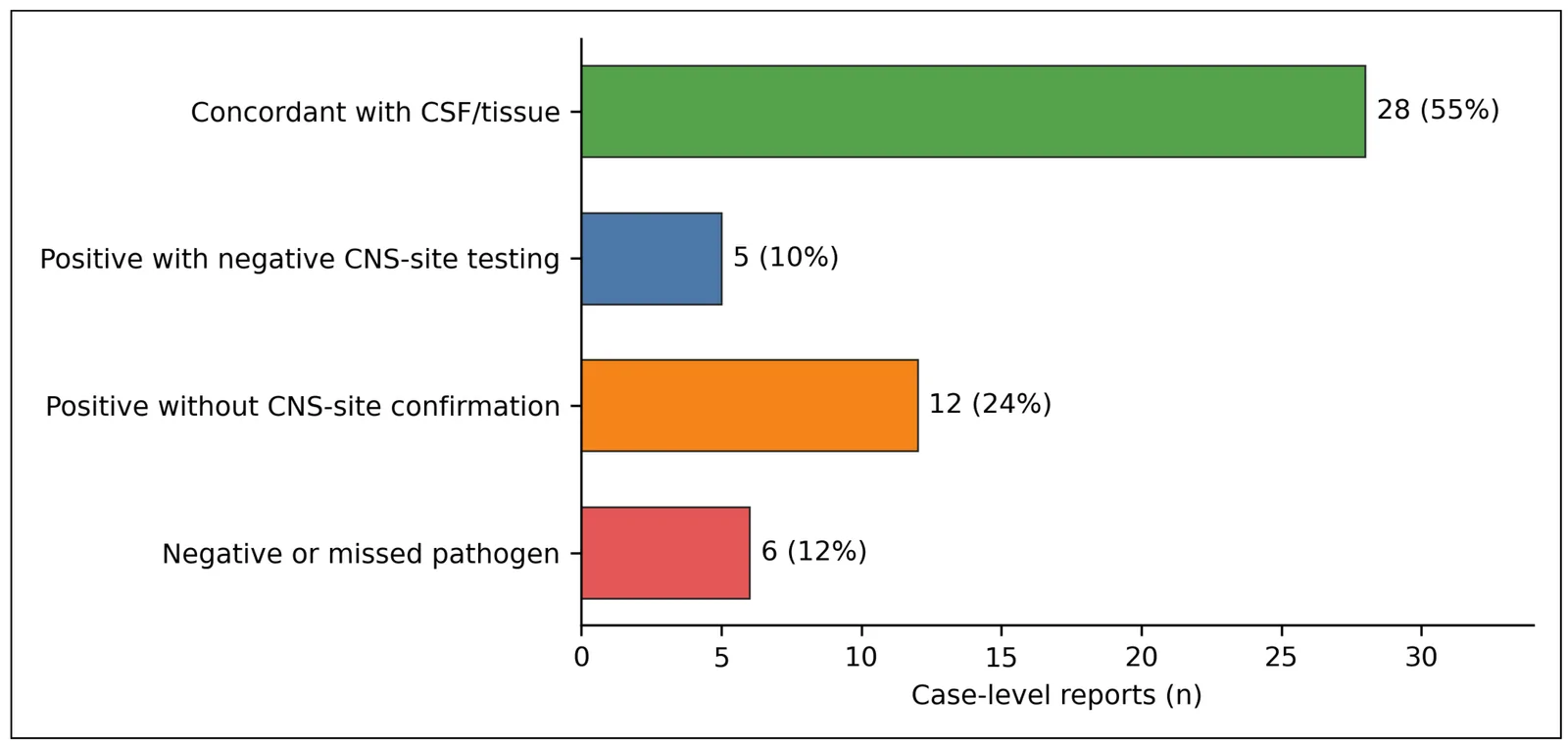
Relation of the plasma or serum result to CNS-site testing.

Per-patient findings of the 51 small series and case reports are reported in Table 4. The principal organisms detected in plasma or serum were bacteria in 15 reports, fungi or moulds in 10, free-living amoebae in 8, viruses in 5, other parasites in 4, and mycobacteria in 3, while 6 reports described a negative or missed result (Figure 3). The relationship of the plasma or serum result to CNS-site testing is shown in Figure 4: the result was concordant with CSF or tissue in 28 reports (55%), positive when CNS-site testing was negative in 5 (10%; *Cladophialophora* ventriculitis, *Balamuthia* and *Acanthamoeba* encephalitis, congenital tularemia, and *Scedosporium* brain abscess), positive without CNS-site confirmation in 12 (24%), and negative or missed the pathogen in 6 (12%; Toscana virus, tick-borne and yellow-fever vaccine virus encephalitis tested by DNA-inclusive workflows aside, *Angiostrongylus* serum, group B streptococcal meningitis, and *Rhizomucor* brain abscess). Reported turnaround for commercial testing ranged from under 24 h to 5 days, with plasma results preceding culture or tissue PCR by 6 days to 6 weeks in 7 reports, whereas one pediatric *Balamuthia* result took 11 days and one adult result returned postmortem. Incidental co-detections (*Streptococcus macedonicus*, HHV-7, *Acinetobacter haemolyticus*, two colonizers with *Trichosporon*) and chromosomally integrated HHV-6 were explicitly noted as interpretive hazards. Management changes attributed to the plasma or serum result were reported in 39 case-level reports, most often narrowing or initiation of organism-directed therapy; deaths were reported in 16. Among the 43 single case reports, the result was concordant in 24, positive with negative CNS-site testing in 5, unconfirmed in 10, and negative or missed in 4; among the 8 small series, it was concordant in 4, unconfirmed in 2, and negative or missed in 2, with no plasma-only detection. All five plasma-only detections therefore arise from single case reports, so the 55% concordance reflects what has been published rather than diagnostic yield in an unselected population. Of the 28 concordant reports, 20 were rated low or low–moderate concern, whereas none of the 12 unconfirmed reports was rated better than moderate. Confirmation occurred in cerebrospinal fluid alone in 14, in brain or meningeal tissue or at autopsy alone in 4, in both in 1, and was not stated clearly enough to classify in 9. Two reports coded concordant were re-checked against their sources: in the Cladophialophora case [69] serial lumbar cerebrospinal fluid cultures were negative but fourth-ventricle fluid confirmed the organism, and in the Listeria case [84] cerebrospinal fluid culture, three multiplex panels, and cerebrospinal fluid metagenomics were negative while brain-biopsy broad-range polymerase chain reaction confirmed it seven days after the plasma result; both codes stand. Excluding the four abstract-level case reports leaves 47 reports with 26 concordant, 4 plasma-only, 11 unconfirmed, and 6 negative or missed, with no proportion moving by more than 1.3 percentage points; the fifth abstract-level report is the only dedicated meningitis cohort, whose 36%, 50%, and 57% could not be verified against a full text.

### 3.6. Risk of Bias and Overlap

Table 5 gives the design and overall concern for each study. Cohorts were mostly retrospective with reference standards that incorporated the index test and with CNS subgroups too small for stable estimates; 8 were rated moderate, 4 moderate–high, and 6 high among the 18 denominator-based cohorts. Among the 51 case-level reports, 3 were rated low, 24 low–moderate (independent CNS-site or autopsy confirmation), 14 moderate, 1 moderate–high, and 9 high, the latter comprising abstract-level extractions, uncertain causality, or unconfirmed infection. The 2026 infant cohort was an expanded re-adjudication of infants within the 2025 1000-test cohort from the same institution and was not counted as an independent estimate. One further overlap was identified: the 2021 report of CNS invasive aspergillosis in a liver transplant recipient [65] and the 2022 single-centre clinical-impact cohort [34] share a first author and institution; the cohort covers 80 patients tested between May 2019 and February 2021, and its reference list cites that case report, so the two are not treated as independent and the 51 case-level reports describe at most 50 patients independent of the included cohorts. Neither source states the overlap explicitly, so this is an inference from institution, enrolment window, and citation. No other patient overlap was identified.

## 4. Discussion

### 4.1. Principal Findings

Across 69 studies, plasma or serum metagenomic sequencing behaved as a complementary rather than a replacement test for CNS infection. Two patterns dominate the evidence. First, in unselected clinical-impact cohorts the CNS subgroups showed low positive impact, with no impactful result among adult meningoencephalitis tests in the largest series and impact confined to a fifth of pediatric tests [42,43,46]; the single dedicated meningitis cohort found plasma testing less productive than conventional testing yet additive when combined [39]. Second, in case-level reports the plasma or serum result was independently confirmed at the CNS site in just over half, and in a small but clinically important group it was the only positive test after negative CSF or tissue studies, almost always in disseminated or angioinvasive fungal, amoebic, or fastidious bacterial disease [75,82,87,97,99]. The prespecified hypothesis was therefore supported: benefit is concentrated where organisms shed into blood and where CNS-site sampling has failed, not in unselected meningoencephalitis.

### 4.2. Comparison with Previous Literature

The findings are consistent with, and refine, syndrome-agnostic evidence. The multicenter study that introduced the clinical-impact framework reported positive impact in 7.3% of tests overall [20], and the meta-analysis of plasma cell-free mNGS reported pooled agreement without CNS stratification [25]; the CNS subgroups collated here fall at or below those cohort-wide figures. Conversely, CSF-based reviews and the landmark CSF mNGS study show that sequencing of the infected compartment yields diagnoses missed by conventional testing in a meaningful minority of meningoencephalitis cases [14,26,27], and the paired-specimen studies in this review confirm that CSF outperforms blood for diffuse meningeal and viral disease [31,35,38]. A large laboratory series describing the spectrum of organisms detected in plasma across more than 15,000 patients could not contribute CNS-specific data [19], underscoring that indication-level reporting is rare.

### 4.3. Mechanistic and Clinical Interpretation

Several biological factors explain the observed pattern. Circulating microbial cell-free DNA depends on organism burden, tissue invasion, vascular access, and host clearance [17,18]; angioinvasive moulds, free-living amoebae with vasculitic spread, *Listeria* rhombencephalitis, and disseminated infection with endocarditis therefore produced strong plasma signals even when CSF was sterile [73,81,84,92], whereas an encapsulated *Rhizomucor* abscess, tuberculous meningitis with signals below the reporting threshold, and early sampling in cerebral toxoplasmosis produced false-negative results [32,52,86,88]. Assay chemistry matters: DNA-only commercial testing cannot detect RNA viruses, which explains the negative results in Powassan, Toscana, and tick-borne encephalitis cases in which research RNA workflows or PCR were positive [44,49,58]. Interpretation is further complicated by commensal co-detections, reporting thresholds that are not harmonized across assays, and latent or chromosomally integrated viral DNA, as illustrated by HHV-6 encephalitis with a virus-to-cell ratio consistent with integration [22,74]. Reported turnaround of one to five days can precede culture or tissue PCR by days to weeks, which is where earlier organism-directed therapy or avoidance of brain biopsy was described [67,78,84], although delayed or postmortem results provided explanation rather than benefit [81,82].

### 4.4. Heterogeneity

The evidence base is heterogeneous in ways that preclude pooling. Populations ranged from neonates to transplant recipients; index tests included one commercial DNA assay and numerous laboratory-developed DNA or RNA workflows with different thresholds and units; reference standards ranged from CDC PCR and autopsy to retrospective chart adjudication that incorporated the index test; and clinical-impact definitions differed between institutions. Case reports are subject to strong publication bias toward unusual successful diagnoses, which likely inflates the apparent frequency of plasma-only detections, while the exclusion of 34 reports that described sequencing of “blood” without specifying the fraction, most from China, may have removed genuine plasma cell-free sequencing data and biases the geographic distribution toward United States commercial testing. Two further sources of bias deserve mention. There is no standardized threshold for positivity in plasma metagenomic sequencing: thresholds are set by each vendor or laboratory, are organism-dependent for the commercial assay, and are unstated in several reports, so marginal results are not comparable between platforms and no quantitative cut-off can be recommended. Separately, a single commercial assay is named in 41 of the 69 reports, and commercial sequencing in this field is often performed, funded, or co-authored with the manufacturer. Neither QUADAS-2 nor the case-level domains contain a sponsorship item, and sponsorship bias in diagnostic research operates through choice of comparator, selective reporting of favourable subgroups, and framing of indeterminate results as positive, all of which would inflate apparent yield.

### 4.5. Clinical Implications

Plasma or serum sequencing appears most defensible when the probability of CNS infection remains substantial after standard evaluation, when disseminated, angioinvasive, or fastidious pathogens are plausible, when prior therapy may have sterilized cultures, or when lumbar puncture or biopsy is unsafe or nondiagnostic. A positive result should be checked for biological plausibility, an extracranial source, organism burden relative to the reporting threshold, and orthogonal confirmation; a negative result should not end evaluation when localized low-burden disease, tuberculosis, toxoplasmosis, or an RNA virus remains possible. Routine ordering in unselected adult meningoencephalitis or brain abscess is not supported by the cohort data, and diagnostic stewardship frameworks are appropriate [21].

### 4.6. Methodological Implications and Future Research

Future studies should enrol consecutive patients within prespecified CNS syndromes, collect plasma and CSF at standardized times relative to antimicrobial exposure, use a composite reference standard independent of the index test, report full 2 × 2 data including indeterminate results, prespecify clinical-impact definitions, report all detections including those judged incidental, state the blood fraction and assay chemistry explicitly, and identify shared cohorts. RNA-inclusive workflows deserve evaluation in encephalitis. A prospective comparative cohort or strategy trial of CSF-based testing with versus without plasma sequencing could determine whether time to correct therapy or unnecessary treatment is altered.

### 4.7. Strengths and Limitations

Strengths include explicit PICOS criteria, a reproducible search with documented strings, full-text verification of every extracted value, separation of yield from clinical impact, explicit handling of overlap, and equal weighting of negative and discordant results. Limitations are substantial. The database search was confined to PubMed/MEDLINE because Embase, Scopus, Web of Science, the Cochrane Library, and Europe PMC could not be accessed, so non-MEDLINE reports may have been missed despite supplementary searching. Screening, extraction, and appraisal were performed independently by two authors with disagreements resolved by discussion, but no formal agreement statistic was calculated. The review was not registered prospectively, so the eligibility criteria, outcome definitions, and subgroup choices cannot be verified as prespecified. Thirteen reports could not be retrieved and five were extracted at abstract level. The broad syndrome definition, which included spinal epidural and endocarditis-associated intracranial infection, increases clinical heterogeneity, and several included reports rest on weak CNS ascertainment. Qualitative risk-of-bias labels are not validated scores, and no meta-analysis or certainty rating was possible.

## 5. Conclusions

Untargeted metagenomic sequencing of plasma or serum identified a plausible pathogen in selected central nervous system infections without lumbar puncture or biopsy, and in five published cases it was the only positive test after negative cerebrospinal fluid or tissue studies, each in disseminated fungal, amoebic, or fastidious bacterial disease. In unselected meningoencephalitis, its documented clinical impact was low and it was less productive than conventional testing, while DNA-only assays missed RNA viruses, compartmentalized low-burden disease, and chromosomally integrated human herpesvirus 6. Plasma or serum sequencing is therefore an adjunct for a defined clinical question rather than a replacement for cerebrospinal fluid or tissue diagnostics, and prospective syndrome-specific studies with independent reference standards are required before accuracy can be quantified.

## Figures and Tables

**Table 1:** Characteristics of included studies.

Study	Country	Design	CNS n/N	Age Group	Immune Status	CNS Syndrome	Age Group (WHO/MeSH) †	Immune Status (Standardized) †
Haston 2020 [31]	USA	Prospective cohort	20/20	Children	Immunocompetent	Encephalitis	Children	Immunocompetent
Niles 2020 [32]	USA	Retrosp. cohort	3/60	Children	62% IC	Meningitis/encephalitis	Children	Mixed (~37/60, 62%)
Liao 2021 [33]	China	Prospective cohort	31/32	Children 0–14 y	NR	Bacterial meningitis	Children ‡	NR
Shishido 2022 [34]	USA	Retrosp. cohort	10/80	Adults	56% IC	Meningoencephalitis	Adults	Mixed (~45/80, 56%)
Chen 2022 [35]	China	Prospective cohort	27/168	Neonates	NR	Meningitis + BSI	Neonates	NR
Han 2023 [36]	China	Retrosp. cohort	1/147	Adults	64% IC	Suspected CNS infection	Adults	Mixed (~94/147, 64%)
Linder 2023 [37]	USA	Retrosp. cohort	3/71	Adults	35% IC	Brain abscess	Adults	Mixed (~25/71, 35%)
Fan 2023 [38]	China	Cross-sectional	113/113	Children	NR	Meningitis/encephalitis	Children	NR
Duster et al. 2024 [39]	USA	Retrosp. case series	28/28	Children	NR	Meningitis	Children	NR
Lehman 2024 [40]	USA	Retrosp. cohort	4/104	Children	100% IC	CNS infection	Children	Immunocompromised (104/104)
Pang et al. 2024 [41]	China	Multicenter retrosp.	10/653	NR	NR	Meningitis	NR	NR
Kaur 2025 [42]	USA	Retrosp. cohort	78/1000	Adults + children	66%/37% IC	Meningoencephalitis; CNS lesion	Mixed	Mixed (66%/37% as published)
Jung 2025 [43]	USA	Retrosp. cohort	19/88	Adults	NR	ME; abscess; vasculitis	Adults	NR
Ranganath 2025 [44]	USA	Retrosp. cohort (FUO)	3/176	Adults	51% IC	Powassan encephalitis	Adults	Mixed (~90/176, 51%)
Pithia 2026 [45]	USA	Retrosp. cohort	13/95	Infants	NR	ME; CNS lesion; spinal abscess	Infants	NR
Osborn 2026 [46]	USA	Retrosp. cohort	9/120	Adults	58% IC	Neurologic indication	Adults	Mixed (~70/120, 58%)
Berger 2026 [47]	USA	Retrosp. cohort (mould+)	3/30	Adults	Mixed	Focal CNS lesions (aspergillosis)	Adults	Mixed
Centeno 2026 [48]	USA	Retrosp. series	4/5	Adults	4/5 IC	CNS toxoplasmosis	Adults	Mixed (4/5)
Lipowski 2017 [49]	Poland	Case series (3)	3/3	Adults	100% SOT	TBEV encephalitis	Adults	Immunocompromised
Horiba 2021 [50]	Japan	Case series (2)	2/2	Infants	Immunocompetent	GBS meningitis	Infants	Immunocompetent
Gould 2023 [51]	USA	Outbreak series	4/6	Adults	100% SOT	YFV-17D meningoencephalitis	Adults	Immunocompromised
Li 2024 [52]	USA	Case series (3)	3/3	Children	Immunocompetent	Tuberculous meningitis	Children	Immunocompetent
Griffin 2024 [53]	USA	Outbreak investigation	1/6	Adult	Immunocompetent	Fungal meningitis (donor)	Adults	Immunocompetent
Chance 2024 [54]	USA	Case series (3)	3/3	Children	Immunocompetent	Eosinophilic ME	Children	Immunocompetent
Vazquez 2025 [55]	USA	Case series (9)	1/9	Child	Immunocompetent	Encephalitis	Children	Immunocompetent
Ahmed 2025 [56]	USA	Case series (10)	2/10	Toddlers	Immunocompetent	Spinal epidural phlegmon	Children	Immunocompetent
Jacobs 2016 [57]	UK	Case report	1	Adult	Immunocompetent	Ebola meningoencephalitis	Adults	Immunocompetent
Tschumi 2019 [58]	Switzerland	Case report	1	Adult	Immunocompetent	Toscana virus meningitis	Adults	Immunocompetent
Feng 2020 [59]	China	Case report	1	Adult	Immunocompetent	Eosinophilic ME	Adults	Immunocompetent
Kalyatanda 2020 [60]	USA	Case report	1	Adult	Immunocompetent	GAE	Adults	Immunocompetent
Asif 2021 [61]	USA	Case report	1	Adult	Immunocompetent	Bacterial meningitis	Adults	Immunocompetent
Brewer 2021 [62]	USA	Case report	1	Adolescent	HCT	Cerebral toxoplasmosis	Children	Immunocompromised
Huang 2021 [63]	China	Case report	1	Child	Immunocompetent	PAM	Children	Immunocompetent
Kattner 2021 [64]	Germany	Case report	1	Adult	NR	Cerebral aspergillosis	Adults	NR
Shishido 2021 [65]	USA	Case report	1	Adult	Liver transplant	Fungal brain abscess	Adults	Immunocompromised
Srichawla 2022 [66]	USA	Case report	1	Adult	Immunocompetent	Multiple brain abscesses	Adults	Immunocompetent
Arrighi-Allisan 2022 [67]	USA	Case report	1	Adult	Liver transplant	Multiple brain abscesses	Adults	Immunocompromised
Abe 2022 [68]	USA	Case report	1	Infant	Immunocompetent	Eosinophilic meningoencephalomyelitis	Infants	Immunocompetent
Boguniewicz 2022 [69]	USA	Case report	1	Child	NR	Meningitis/ventriculitis/cerebritis	Children	NR
Zhou 2022 [70]	China	Case report	1	Child	Immunocompetent	PAM	Children	Immunocompetent
Peng 2022 [71]	China	Case report	1	Adult	Immunocompetent	GAE	Adults	Immunocompetent
Karim 2022 [72]	USA	Case report	1	Adolescent	Immunocompetent	Spinal epidural abscess	Children	Immunocompetent
Traver 2024 [73]	USA	Case report	1	Adult	Immunocompetent	Intracranial mycotic aneurysm	Adults	Immunocompetent
Blackbourn 2024 [74]	USA	Case report	1	Adult	Immunocompetent	Encephalitis	Adults	Immunocompetent
Whitman 2024 [75]	USA	Case report	1	Adult	Renal transplant	Ventriculitis/cerebritis	Adults	Immunocompromised
Lin 2024 [76]	China	Case report	1	Child	Immunocompetent	PAM	Children	Immunocompetent
Shan 2024 [77]	China	Case report	1	Adult	Immunocompetent	SFTSV encephalitis	Adults	Immunocompetent
Hennis 2024 [78]	USA	Case report	1	Adult	Immunocompetent	Brain abscesses (IE)	Adults	Immunocompetent
Nirmal 2024 [79]	USA	Case report	1	Adult	NR	Cerebral abscesses (IE)	Adults	NR
Chetrit 2024 [80]	USA	Case report	1	Adult	NR	Infectious CNS vasculitis/aneurysm	Adults	NR
Aboubechara et al. 2025 [81]	USA	Case report	1	Child	Immunocompetent	GAE with CNS vasculitis	Children	Immunocompetent
Edminster 2025 [82]	USA	Case report	1	Adult	Immunocompetent	GAE	Adults	Immunocompetent
Karatela 2025 [83]	USA	Case report	1	Adult	Rituximab	Aseptic meningitis	Adults	Immunocompromised
Tran 2025 [84]	USA	Case report	1	Adult	Immunocompetent	Rhombencephalitis	Adults	Immunocompetent
Bristol 2025 [85]	USA	Case report	1	Adult	Immunocompetent	Meningitis, vasculitis, abscess	Adults	Immunocompetent
Valencia Uribe 2025 [86]	USA	Case report	1	Adult	Lymphoma/HLH	Toxoplasmic encephalitis	Adults	Immunocompromised
Nelson 2025 [87]	USA	Case report	1	Neonate	Neonate	Neuroinvasive tularemia	Neonates	Immunocompetent
El Atrache 2025 [88]	USA	Case report	1	Adolescent	Leukemia	Brain abscess (mucormycosis)	Children	Immunocompromised
Phillips 2025 [89]	USA	Case report	1	Infant	Chemotherapy	Fungal meningitis, mycotic aneurysms	Infants	Immunocompromised
Chatterjee 2025 [90]	USA	Case report	1	Adult	Multivisceral transplant	Brain mass (aspergillosis)	Adults	Immunocompromised
Cohodes 2025 [91]	USA	Case report	1	Adult	Kidney transplant	Skull-base TB with cerebral infarct	Adults	Immunocompromised
Ladines-Lim 2025 [92]	USA	Case report	1	Adult	Biologics/steroids	Intracranial abscess (IE)	Adults	Immunocompromised
Alhaj 2025 [93]	USA	Case report	1	Adult	HLH therapy	Suspected fungal meningitis	Adults	Immunocompromised
Jiang 2026 [94]	China	Case report	1	Adult	NR	Meningoencephalitis	Adults	NR
Peng 2026 [95]	China	Case report	1	Adult	Immunosuppressed	Rhino-orbito-cerebral mucormycosis	Adults	Immunocompromised
Koirala 2026 [96]	USA	Case report	1	Child	Immunocompetent	Ruptured infectious aneurysm	Children	Immunocompetent
Wong 2026 [97]	USA	Case report	1	Adult	Lung transplant	Brain abscesses (IE)	Adults	Immunocompromised
Kaira 2026 [98]	USA	Case report	1	Adult	Immunocompetent	Brain abscess, ventriculitis	Adults	Immunocompetent
Wilson 2026 [99]	USA	Case report	1	Adult	Kidney–pancreas transplant	GAE	Adults	Immunocompromised

Abbreviations: BSI, bloodstream infection; CNS, central nervous system; FUO, fever of unknown origin; GAE, granulomatous amoebic encephalitis; GBS, group B streptococcus; HCT, hematopoietic cell transplant; HLH, hemophagocytic lymphohistiocytosis; IC, immunocompromised or immunosuppressed; IE, infective endocarditis; ME, meningoencephalitis; N, source denominator (tests or patients); NR, not reported; PAM, primary amoebic meningoencephalitis; Retrosp., retrospective; SFTSV, severe fever with thrombocytopenia syndrome virus; SOT, solid-organ transplant; TB, tuberculosis; TBEV, tick-borne encephalitis virus; y, years; YFV-17D, yellow fever vaccine virus 17D. CNS n/N gives the number of CNS-syndrome patients or tests over the source denominator; single case reports are shown as 1. † Age standardized to the WHO/MeSH categories (neonates 0–28 d; infants 29 d to <1 y; children 1 to <18 y; adults ≥ 18 y), with the verbatim value retained in the preceding column. Immune status is a mutually exclusive variable; counts prefixed ~ were derived from the published percentage and the cohort denominator N and are not values reported in the source. ‡ Reported only as children aged 0–14 y, a range that also spans the neonatal and infant categories.

**Table 2:** Index tests and reference standards.

Study	Specimen	Assay	Reference Standard	Source
Haston 2020 [31]	Serum/plasma	Research mNGS (USAMRIID)	CSF mNGS, CSF PCR, clinical	Full text
Niles 2020 [32]	Plasma	Karius	Conventional ≤ 7 d	Full text
Liao 2021 [33]	Plasma	Research mNGS (WGA, Illumina)	Culture/conventional; CSF mNGS	Full text
Shishido 2022 [34]	Plasma	Karius	Conventional + adjudication	Full text
Chen 2022 [35]	Plasma	Research mNGS (Hugobiotech)	Culture/PCR; final diagnosis	Full text
Han 2023 [36]	Plasma	In-house cfDNA/RNA mNGS	Culture/PCR + adjudication	Full text
Linder 2023 [37]	Plasma	Karius	Culture, 16S PCR, adjudication	Full text
Fan 2023 [38]	Serum	Research total-NA mNGS	CSF mNGS; PCR confirmation	Full text
Duster et al. 2024 [39]	Plasma	Cell-free plasma NGS	Conventional testing	Abstract
Lehman 2024 [40]	Plasma	Karius	Conventional ≤ 30 d	Full text
Pang et al. 2024 [41]	Plasma	PMseq (BGI)	Composite clinical	Full text
Kaur 2025 [42]	Plasma	Karius	Standard tests + panel adjudication	Full text
Jung 2025 [43]	Plasma	Karius	Conventional + adjudication	Full text
Ranganath 2025 [44]	Plasma	Karius	CSF panels/PCR/serology	Full text
Pithia 2026 [45]	Plasma	Karius	Conventional + adjudication	Full text
Osborn 2026 [46]	Plasma	Karius	Composite + consensus adjudication	Full text
Berger 2026 [47]	Plasma	Karius Spectrum	EORTC/MSGERC; sterile-site culture	Full text
Centeno 2026 [48]	Plasma	Karius	CSF PCR, serology, biopsy	Full text
Lipowski 2017 [49]	Serum	Research RNA NGS	RT-PCR; brain/CSF NGS	Full text
Horiba 2021 [50]	Serum	Research mNGS (PATHDET)	CSF NGS; culture	Full text
Gould 2023 [51]	Serum	Research mNGS (UCSF/CDC)	RT-PCR, serology, tissue mNGS	Full text
Li 2024 [52]	Plasma	Karius (addended)	CSF culture/PCR, T-SPOT	Full text
Griffin 2024 [53]	Plasma	Research mNGS (UCSF)	CSF panfungal PCR	Full text
Chance 2024 [54]	Plasma	Karius	CDC CSF PCR	Full text
Vazquez 2025 [55]	Plasma	Karius-type mcfDNA	Serology; CSF panel negative	Full text
Ahmed 2025 [56]	Plasma	Karius	MRI; cultures negative	Full text
Jacobs 2016 [57]	Plasma	Research mNGS	RT-PCR (CSF/plasma), culture	Full text
Tschumi 2019 [58]	Serum	Research mNGS (VirMet)	RT-qPCR (CSF, serum)	Full text
Feng 2020 [59]	Serum	mNGS (Hugobiotech)	CSF mNGS/qPCR, ELISA	Full text
Kalyatanda 2020 [60]	Plasma	Karius	Brain biopsy PCR/IHC	Full text
Asif 2021 [61]	Plasma	Karius	Cultures, CSF panel (negative)	Full text
Brewer 2021 [62]	Plasma	Karius	CSF PCR	Full text
Huang 2021 [63]	Plasma	mNGS (Sagene)	CSF mNGS; PCR/Sanger	Full text
Kattner 2021 [64]	Plasma	DISQVER (Noscendo)	Biopsy; CSF NGS	Abstract
Shishido 2021 [65]	Plasma	Karius	NR (abstract)	Abstract
Srichawla 2022 [66]	Plasma	Plasma mcfDNA NGS (vendor not named)	Cultures negative; imaging	Full text
Arrighi-Allisan 2022 [67]	Plasma	Karius	Biopsy histology/culture	Full text
Abe 2022 [68]	Plasma	Karius	CSF eosinophilia; exposure	Full text
Boguniewicz 2022 [69]	Plasma	Plasma mNGS	Ventricular fluid (confirmed)	Abstract
Zhou 2022 [70]	Plasma	mNGS (Hugobiotech)	PCR/Sanger; CSF smear	Full text
Peng 2022 [71]	Serum	NGS (Vision Medicals)	Brain histology; tissue NGS	Full text
Karim 2022 [72]	Plasma	Plasma mcfDNA NGS	Surgical cultures	Full text
Traver 2024 [73]	Plasma	Karius	Valve 16S NGS	Full text
Blackbourn 2024 [74]	Plasma	Karius	CSF/blood HHV-6 PCR	Full text
Whitman 2024 [75]	Plasma	Karius	CSF NGS/culture (negative)	Full text
Lin 2024 [76]	Plasma	Research mNGS	CSF mNGS; PCR	Full text
Shan 2024 [77]	Serum	mNGS	CSF mNGS	Full text
Hennis 2024 [78]	Plasma	Karius	Cultures negative; imaging	Full text
Nirmal 2024 [79]	Plasma	Karius	Cultures negative	Full text
Chetrit 2024 [80]	Plasma	Karius	Angiography; cultures negative	Full text
Aboubechara et al. 2025 [81]	Plasma	Karius	CDC CSF PCR	Full text
Edminster 2025 [82]	Plasma	Plasma mNGS	Autopsy; tissue qPCR	Full text
Karatela 2025 [83]	Plasma	Karius	CSF broad-range PCR	Full text
Tran 2025 [84]	Plasma	Karius	Brain-tissue PCR	Full text
Bristol 2025 [85]	Plasma	Karius	Skull-base biopsy; tissue PCR	Full text
Valencia Uribe 2025 [86]	Plasma	Karius	Brain biopsy IHC	Full text
Nelson 2025 [87]	Plasma	Karius	Maternal tissue IHC/16S; CSF negative	Full text
El Atrache 2025 [88]	Plasma	Karius	Biopsy fungal PCR	Full text
Phillips 2025 [89]	Plasma	Karius	CSF mNGS	Full text
Chatterjee 2025 [90]	Plasma	Karius Spectrum	Prior nasal culture; galactomannan	Full text
Cohodes 2025 [91]	Plasma	Karius Spectrum	Tissue PCR; culture	Full text
Ladines-Lim 2025 [92]	Plasma	Karius	Brain-tissue culture	Full text
Alhaj 2025 [93]	Plasma	Karius	Cultures/serology negative	Full text
Jiang 2026 [94]	Plasma	Plasma mNGS	Serology; CSF mNGS failed	Full text
Peng 2026 [95]	Serum	mNGS (IDseq)	CSF mNGS; imaging	Full text
Koirala 2026 [96]	Plasma	Karius	Cultures negative (3 sets)	Full text
Wong 2026 [97]	Plasma	Karius	Brain biopsy culture; CSF mNGS	Full text
Kaira 2026 [98]	Serum	Karius	CSF 16S PCR	Full text
Wilson 2026 [99]	Plasma	Plasma mcfDNA NGS	Postmortem	Abstract

Abbreviations: 16S, 16S ribosomal RNA gene; BGI, BGI Genomics; CDC, United States Centers for Disease Control and Prevention; cfDNA, cell-free DNA; CSF, cerebrospinal fluid; d, days; DISQVER, DISQVER plasma cfDNA assay (Noscendo); ELISA, enzyme-linked immunosorbent assay; EORTC/MSGERC, European Organisation for Research and Treatment of Cancer/Mycoses Study Group Education and Research Consortium; IDseq, IDseq mNGS workflow; IHC, immunohistochemistry; Karius, Karius plasma microbial cell-free DNA test; Karius Spectrum, Karius Spectrum plasma mcfDNA test; mcfDNA, microbial cell-free DNA; mNGS, metagenomic next-generation sequencing; NA, nucleic acid; NGS, next-generation sequencing; NR, not reported; PATHDET, PATHDET metagenomic pipeline; PCR, polymerase chain reaction; PMseq, pathogen metagenomic sequencing (BGI); qPCR, quantitative PCR; RT-PCR, reverse-transcription PCR; T-SPOT, T-SPOT.TB interferon-γ release assay; UCSF, University of California, San Francisco; USAMRIID, United States Army Medical Research Institute of Infectious Diseases; VirMet, VirMet viral metagenomics pipeline; WGA, whole-genome amplification. Source indicates whether values were verified in the full text or, when inaccessible, extracted from the published abstract. Specimen records the biological fraction only; one report specified a cell-stabilizing blood collection tube, which limits dilution of microbial cell-free DNA by lysed-cell DNA, and the remainder did not state the tube type. Karius denotes the commercial plasma microbial cell-free DNA assay and Karius Spectrum its later generation; DISQVER, PMseq, Hugobiotech, Sagene, and Vision Medicals are other commercial services; entries naming a pipeline are academic research workflows; Plasma mNGS, Plasma mcfDNA NGS, Cell-free plasma NGS, and mNGS denote laboratory-developed workflows the source did not name, which are not assumed equivalent to one another or to the commercial assay. Srichawla 2022 [66] names no commercial platform and reports in molecules per microlitre, so it is recorded as vendor not named; Vazquez 2025 [55] retains its extracted label.

**Table 3:** CNS-specific findings in denominator-based cohorts.

Study	Plasma/Serum Finding	CNS-Subgroup Result	TAT	Management	Outcome
Haston 2020 [31]	3 pts: *M. bovis* (serum), bocavirus, TMV (plasma)	Serum/plasma detections 3/20; none additional to CSF	~3 d	None (research)	1 death (Balamuthia)
Niles 2020 [32]	*M. tuberculosis* and HSV-1 missed	CNS: 0/3 pathogens detected (2 false-negative)	mNGS 4.0 d vs. CT 1.8 d	None for CNS	NR
Liao 2021 [33]	*K. pneumoniae*, *E. coli* signatures	Blood–CSF dominant-species concordance 9/9 (*K. pneumoniae* group) and 9/17 (other)	NR	NR	All discharged improved
Shishido 2022 [34]	NR by CNS case	Positive impact 2/10 (20%)	NR	Per impact score	NR
Chen 2022 [35]	*K. pneumoniae*, *E. coli*, parechovirus	Paired: blood 19/27 vs. CSF 20/27 positive; agreement with final dx 10/27 vs. 14/27	NR	Therapy changed 86/117 (cohort)	80/86 recovered
Han 2023 [36]	NR	mNGS 1/1 vs. conventional 0/1; positive impact 1/1	Median 28 h	Positive impact	NR
Linder 2023 [37]	Discordant: 216 MPM organism vs. *S. intermedius* on biopsy	Impact 0/3; 1 discordant brain lesion	NR	None	NR
Fan 2023 [38]	CV-A2, adenovirus B	Serum viral pathogen 3/113 vs. CSF 9/113; combined 12/113; no paired concordance	NR	NR	NR
Duster et al. 2024 [39]	NR	Pathogen: plasma 36% vs. conventional 50%; combined 57%	NR	Adjunct	NR
Lehman 2024 [40]	*T. gondii* 5081 MPM	Impact 1/4	3–5 d	Targeted therapy	NR
Pang et al. 2024 [41]	Class-level only	Plasma positive 4/10	NR	NR	NR
Kaur 2025 [42]	NR by CNS case	ME impact: adults 0/24, children 5/24; CNS lesion: adults 2/26, children 1/4	NR	Per impact score	NR
Jung 2025 [43]	HSV-1; *S. boydii*	Impact: ME 1/10, abscess 0/5, vasculitis 1/4	Median 3 d	Per impact score	NR
Ranganath 2025 [44]	Negative (RNA virus)	Plasma positive 0/3	NR	None	NR
Pithia 2026 [45]	*H. influenzae* (1 case)	1 CNS positive-impact case; overall positive 5/95	NR	1 new diagnosis	NR
Osborn 2026 [46]	NR	Impact 1/9	NR	Per impact score	NR
Berger 2026 [47]	*A. fumigatus* ×3 (62, 62, <LOQ MPM)	True positive 3/3	41–90 h	Change in 1/3	1 death
Centeno 2026 [48]	*T. gondii* ×4 (392–955 MPM)	Plasma positive 4/4; CSF PCR 1/2	Median 3 d	Initiated/supported therapy	2 LTACH, 1 hospice, 1 home

Abbreviations: *A. fumigatus*, *Aspergillus fumigatus*; CNS, central nervous system; CSF, cerebrospinal fluid; CT, conventional testing; CV-A2, coxsackievirus A2; d, days; dx, diagnosis; *E. coli*, *Escherichia coli*; h, hours; *H. influenzae*, *Haemophilus influenzae*; HSV-1, herpes simplex virus 1; *K. pneumoniae*, *Klebsiella pneumoniae*; LOQ, limit of quantification; LTACH, long-term acute care hospital; *M. bovis*, *Mycobacterium bovis*; *M. tuberculosis*, *Mycobacterium tuberculosis*; ME, meningoencephalitis; mNGS, metagenomic next-generation sequencing; MPM, DNA molecules per microliter (as reported); NR, not reported; PCR, polymerase chain reaction; *S. boydii*, *Scedosporium boydii*; *S. intermedius*, *Streptococcus intermedius*; *T. gondii*, *Toxoplasma gondii*; TAT, turnaround time; TMV, tobacco mosaic virus; vs., versus. Numerators and denominators are reproduced from each source; units and thresholds differ between assays and were not harmonized.

**Table 4:** Case-level findings.

Study	Plasma/Serum Finding	Relation to CNS-Site Testing	Result Detail	TAT	Management	Outcome
Lipowski 2017 [49]	TBEV (serum, 2/2 tested)	Concordant	Serum NGS positive 2/2	NR (retrospective)	None	3 deaths
Horiba 2021 [50]	Serum negative (1/1 tested)	Negative/missed	Serum NGS 0/1 vs. CSF NGS 1/1	NR	None	Recovered
Gould 2023 [51]	Serum negative (1/1 tested)	Negative/missed	Serum mNGS 0/1; CSF 1/4; tissue 1/1	NR	None	2/4 died
Li 2024 [52]	MTBC ×3 (subthreshold)	Concordant	Plasma signal 3/3 below standard threshold	1.8–2.7 d	Supported early therapy	NR
Griffin 2024 [53]	*Fusarium solani* complex	Concordant	Plasma positive 1/1 (0.12 RPM)	NR (retrospective)	Recipient prophylaxis	Donor died
Chance 2024 [54]	*A. cantonensis* ×3 (186, 27, 127 MPM)	Concordant	Plasma 3/3 vs. CSF PCR 2/3	NR	Steroids ± albendazole	All survived
Vazquez 2025 [55]	*B. henselae*	Unconfirmed	Plasma positive 1/1	NR	Doxycycline + rifampin	Improved
Ahmed 2025 [56]	*K. kingae* ×2	Unconfirmed	Plasma sole diagnosis 2/2	2.5 d	Tailored antibiotics	NR
Jacobs 2016 [57]	Zaire ebolavirus	Concordant	Plasma positive (Ct 31 vs. CSF 24)	NR	Excluded co-infection	Survived, deficits
Tschumi 2019 [58]	Negative (CSF positive)	Negative/missed	Serum 0 reads; CSF 284 reads	NR	Acyclovir stopped	Recovered
Feng 2020 [59]	Negative (CSF positive)	Negative/missed	Serum negative; CSF *A. cantonensis*	~2 d	None (CSF-directed)	Died
Kalyatanda 2020 [60]	*B. mandrillaris* (649 reads)	Concordant	Plasma sole antemortem detection	48 h	Anti-amoebic therapy	Died d13
Asif 2021 [61]	*C. canimorsus* 11 MPM	Unconfirmed	Plasma sole detection	<24 h	Narrowed to ceftriaxone	Recovered
Brewer 2021 [62]	*T. gondii* 5081 MPM	Concordant	Plasma and CSF PCR concordant	2 d (CSF PCR 4 d)	Therapy initiated	Survived, deficits
Huang 2021 [63]	*N. fowleri* (64 reads)	Concordant	Plasma 64 vs. CSF 326 reads	NR	None (post brain death)	Died
Kattner 2021 [64]	*Aspergillus* spp.	Concordant	Plasma and CSF positive	NR	NR	NR
Shishido 2021 [65]	*Aspergillus* spp.	Unconfirmed	Plasma positive	NR	NR	NR
Srichawla 2022 [66]	*F. nucleatum* 1779; *R. mucilaginosa* 399 MPM	Unconfirmed	Plasma sole detection	NR	Narrowed therapy	Improved
Arrighi-Allisan 2022 [67]	*C. bantiana* 254 MPM	Concordant	Plasma 6 d before culture	3 d	Antifungals added	Died d29
Abe 2022 [68]	*A. cantonensis*	Unconfirmed	Plasma positive d22	NR	Steroids + albendazole	Survived, deficits
Boguniewicz 2022 [69]	*C. bantiana*	Concordant	Plasma positive; lumbar CSF cultures negative; 4th-ventricle fluid confirmed	NR	Surgery + antifungals	Alive 3 y, deficits
Zhou 2022 [70]	*N. fowleri* (1337 reads)	Concordant	Plasma and CSF positive	NR	None (died)	Died
Peng 2022 [71]	*B. mandrillaris* (112 reads)	Concordant	Serum 112, CSF 539, tissue 3723 reads	~2–3 d	Multidrug + surgery	Survived
Karim 2022 [72]	*K. aerogenes*	Concordant	Plasma positive; cultures concordant	NR	Regimen changed; surgery	Resolved 9 mo
Traver 2024 [73]	*B. rochalimae* 47,501 MPM	Concordant	Plasma d4; tissue NGS d36	2 d	Doxycycline-based therapy	Survived
Blackbourn 2024 [74]	HHV-6B; *S. macedonicus* (incidental)	Concordant	Concordant; ciHHV-6 confounds	NR	Foscarnet	Improved
Whitman 2024 [75]	*C. bantiana* 11 MPM	Positive; CNS-site negative	Plasma positive; CSF NGS negative	~2 d	Antifungals started	Died
Lin 2024 [76]	*N. fowleri* (2503 reads)	Concordant	Plasma and CSF positive	~40 h	Amphotericin + rifampicin	Died
Shan 2024 [77]	SFTSV (59 seq)	Concordant	Serum 59 vs. CSF 12 sequences	NR	Foscarnet, IVIG	Recovered
Hennis 2024 [78]	*S. intermedius*	Unconfirmed	Plasma sole detection	NR	Biopsy avoided; narrowed	Resolved
Nirmal 2024 [79]	*S. gordonii*	Unconfirmed	Plasma sole detection	NR	Targeted therapy	Improved
Chetrit 2024 [80]	*P. melaninogenica* 72 MPM	Unconfirmed	Plasma sole detection	NR	Ceftriaxone + immunotherapy	Near-complete recovery
Aboubechara et al. 2025 [81]	*B. mandrillaris*	Concordant	Plasma and CSF PCR concordant	11 d	Multidrug therapy	Died d57
Edminster 2025 [82]	*B. mandrillaris*	Positive; CNS-site negative	Plasma positive; CSF mNGS/PCR negative	Postmortem	None	Died
Karatela 2025 [83]	*L. noguchii*	Concordant	Plasma species-level; CSF PCR genus-level	1 d after CSF PCR	Extended therapy	Resolved
Tran 2025 [84]	*L. monocytogenes*	Concordant	Plasma positive; CSF culture/panels/NGS negative; brain-biopsy PCR confirmed	2 d (tissue PCR 9 d)	Ampicillin-based	Survived, deficits
Bristol 2025 [85]	*A. israelii* 61 MPM; HHV-7 (incidental)	Concordant	Plasma d8; biopsy concordant	~4 d	Drainage; narrowed	Died
Valencia Uribe 2025 [86]	*T. gondii* (repeat test)	Concordant	Initial negative; repeat positive	NR	TMP-SMX	Recovered, deficits
Nelson 2025 [87]	*F. tularensis* 804 MPM	Positive; CNS-site negative	Plasma positive; CSF culture/panel negative	NR	Gentamicin + ciprofloxacin	Well at 15 mo
El Atrache 2025 [88]	Missed *R. pusillus*; *A. haemolyticus* (incidental)	Negative/missed	False negative	NR	None (tissue-led)	Improved
Phillips 2025 [89]	*T. inkin* 2102 MPM; 2 colonizers	Concordant	Plasma d5; CSF mNGS d15 concordant	<5 d	Narrowed to voriconazole	Improved
Chatterjee 2025 [90]	*A. calidoustus* 10,244 MPM	Unconfirmed	Plasma positive; no CNS tissue	NR	Antifungal regimen changed	Died
Cohodes 2025 [91]	*M. tuberculosis*	Concordant	Plasma d31; PCR d33; culture d87	~2 d	Anti-TB therapy	Discharged d60
Ladines-Lim 2025 [92]	*M. chimaera* 3788 MPM	Concordant	Plasma ~2 wk before tissue culture	~2 d	Anti-mycobacterial; surgery	Stable 5 mo
Alhaj 2025 [93]	Negative	Negative/missed	Negative; infection not confirmed	NR	Antimicrobials stopped	Died (HLH)
Jiang 2026 [94]	EBV (8 reads)	Unconfirmed	Plasma positive; CSF uninterpretable	NR	Acyclovir	Recovered
Peng 2026 [95]	*Rhizopus delemar* (71 reads)	Concordant	Serum and CSF positive	NR	Switched to isavuconazole	Deteriorating (AMA)
Koirala 2026 [96]	*G. haemolysans* 2848 MPM	Unconfirmed	Plasma sole detection	Day 5	Ceftriaxone + gentamicin	Recovered
Wong 2026 [97]	*Scedosporium* 63 MPM	Positive; CNS-site negative	Plasma positive; CSF mNGS negative	NR	Voriconazole; biopsy	Near-resolution 4 mo
Kaira 2026 [98]	*A. aphrophilus*	Concordant	Serum and CSF PCR concordant	NR	Narrowed; drainage	Improved
Wilson 2026 [99]	*Acanthamoeba* spp.	Positive; CNS-site negative	Plasma positive; CSF nondiagnostic	NR	Presumptive diagnosis	Died

Abbreviations: *A. aphrophilus*, *Aggregatibacter aphrophilus*; *A. calidoustus*, *Aspergillus calidoustus*; *A. cantonensis*, *Angiostrongylus cantonensis*; *A. haemolyticus*, *Acinetobacter haemolyticus*; *A. israelii*, *Actinomyces israelii*; AMA, against medical advice; *B. henselae*, *Bartonella henselae*; *B. mandrillaris*, *Balamuthia mandrillaris*; *B. rochalimae*, *Bartonella rochalimae*; *C. bantiana*, *Cladophialophora bantiana*; *C. canimorsus*, *Capnocytophaga canimorsus*; ciHHV-6, chromosomally integrated HHV-6; CNS, central nervous system; CSF, cerebrospinal fluid; Ct, cycle threshold; d, days; EBV, Epstein–Barr virus; *F. nucleatum*, *Fusobacterium nucleatum*; *F. tularensis*, *Francisella tularensis*; *G. haemolysans*, *Gemella haemolysans*; h, hours; HHV-6B, human herpesvirus 6B; HHV-7, human herpesvirus 7; HLH, hemophagocytic lymphohistiocytosis; IVIG, intravenous immunoglobulin; *K. aerogenes*, *Klebsiella aerogenes*; *K. kingae*, *Kingella kingae*; *L. monocytogenes*, *Listeria monocytogenes*; *L. noguchii*, *Leptospira noguchii*; *M. chimaera*, *Mycobacterium chimaera*; *M. tuberculosis*, *Mycobacterium tuberculosis*; mNGS, metagenomic next-generation sequencing; mo, months; MPM, DNA molecules per microliter (as reported); MTBC, *Mycobacterium tuberculosis* complex; N, source denominator (tests or patients); *N. fowleri*, *Naegleria fowleri*; NGS, next-generation sequencing; NR, not reported; *P. melaninogenica*, *Prevotella melaninogenica*; PCR, polymerase chain reaction; *R. mucilaginosa*, *Rothia mucilaginosa*; *R. pusillus*, *Rhizomucor pusillus*; RPM, reads per million; *S. gordonii*, *Streptococcus gordonii*; *S. intermedius*, *Streptococcus intermedius*; *S. macedonicus*, *Streptococcus macedonicus*; seq, sequences; SFTSV, severe fever with thrombocytopenia syndrome virus; *T. gondii*, *Toxoplasma gondii*; *T. inkin*, *Trichosporon inkin*; TAT, turnaround time; TB, tuberculosis; TBEV, tick-borne encephalitis virus; TMP-SMX, trimethoprim-sulfamethoxazole; vs., versus; wk, weeks; y, years. Relation to CNS-site testing: concordant = same organism in CSF or tissue; positive; CNS-site negative = plasma/serum positive with negative CSF or tissue testing; unconfirmed = no CNS-site confirmation; negative/missed = plasma/serum negative or causative organism not detected.

**Table 5:** Risk-of-bias appraisal.

Study	Design	Overall Concern	Main reason	Overall Judgement §
Haston 2020 [31]	Prospective cohort	Moderate	Research assay; small; blood in subset	High
Niles 2020 [32]	Retrosp. cohort	High	Retrospective; composite reference; tiny subgroup	High
Liao 2021 [33]	Prospective cohort	High	Microbiome design; WGA bias; no per-patient accuracy	High
Shishido 2022 [34]	Retrosp. cohort	High	Retrospective; no CNS organism data	High
Chen 2022 [35]	Prospective cohort	Moderate	Index test in reference; single centre	High
Han 2023 [36]	Retrosp. cohort	High	One CNS case; incorporation bias	High
Linder 2023 [37]	Retrosp. cohort	Mod–high	Retrospective; tiny subgroup	High
Fan 2023 [38]	Cross-sectional	Moderate	Research assay; no clinical reference	High
Duster et al. 2024 [39]	Retrosp. case series	High	Abstract-level; unverifiable methods	Unclear
Lehman 2024 [40]	Retrosp. cohort	Mod–high	Retrospective; tiny subgroup	High
Pang et al. 2024 [41]	Multicenter retrosp.	High	Baseline-table data only	High
Kaur 2025 [42]	Retrosp. cohort	Moderate	Retrospective adjudication; overlaps Pithia 2026 [45]	High
Jung 2025 [43]	Retrosp. cohort	Mod–high	Retrospective; small cells	High
Ranganath 2025 [44]	Retrosp. cohort (FUO)	Moderate	Retrospective; CNS cases incidental	High
Pithia 2026 [45]	Retrosp. cohort	Moderate	Overlaps Kaur 2025 [42]; CNS impact not itemized	High
Osborn 2026 [46]	Retrosp. cohort	Mod–high	Retrospective; syndrome undefined	High
Berger 2026 [47]	Retrosp. cohort (mould+)	Moderate	Selected on positive test	High
Centeno 2026 [48]	Retrosp. series	Moderate	Selected on positive test	High
Lipowski 2017 [49]	Case series (3)	Low–mod	Strong molecular confirmation	Low
Horiba 2021 [50]	Case series (2)	Moderate	Retrospective research NGS	High
Gould 2023 [51]	Outbreak series	Low–mod	Multilayer confirmation	Low
Li 2024 [52]	Case series (3)	Mod–high	Post hoc threshold	High
Griffin 2024 [53]	Outbreak investigation	Low–mod	Concordant CSF PCR	Low
Chance 2024 [54]	Case series (3)	Low–mod	CDC confirmation in 2/3	Low
Vazquez 2025 [55]	Case series (9)	Moderate	Serologic corroboration only	High
Ahmed 2025 [56]	Case series (10)	Moderate	No microbiologic confirmation	High
Jacobs 2016 [57]	Case report	Low–mod	Concordant PCR	Low
Tschumi 2019 [58]	Case report	Low–mod	Concordant PCR	Low
Feng 2020 [59]	Case report	Low–mod	Concordant CSF testing	Low
Kalyatanda 2020 [60]	Case report	Low–mod	Tissue confirmation	Low
Asif 2021 [61]	Case report	Moderate	No independent confirmation	High
Brewer 2021 [62]	Case report	Low–mod	Concordant CSF PCR	Low
Huang 2021 [63]	Case report	Low–mod	Concordant CSF/PCR	Low
Kattner 2021 [64]	Case report	High	Abstract-level	Unclear
Shishido 2021 [65]	Case report	High	Abstract-level	Unclear
Srichawla 2022 [66]	Case report	High	No site confirmation	High
Arrighi-Allisan 2022 [67]	Case report	Low–mod	Tissue confirmation	Low
Abe 2022 [68]	Case report	Moderate	No CSF PCR confirmation	High
Boguniewicz 2022 [69]	Case report	High	Abstract-level	Unclear
Zhou 2022 [70]	Case report	Low–mod	Concordant PCR/smear	Low
Peng 2022 [71]	Case report	Low–mod	Tissue confirmation	Low
Karim 2022 [72]	Case report	Low–mod	Culture confirmation	Low
Traver 2024 [73]	Case report	Low–mod	Tissue confirmation	Low
Blackbourn 2024 [74]	Case report	High	Causality uncertain (ciHHV-6)	High
Whitman 2024 [75]	Case report	High	No tissue confirmation	High
Lin 2024 [76]	Case report	Low–mod	Concordant CSF	Low
Shan 2024 [77]	Case report	Moderate	No PCR quantification	High
Hennis 2024 [78]	Case report	Moderate	No site confirmation	High
Nirmal 2024 [79]	Case report	Moderate	No site confirmation	High
Chetrit 2024 [80]	Case report	Moderate	No site confirmation	High
Aboubechara et al. 2025 [81]	Case report	Low–mod	CDC confirmation	Low
Edminster 2025 [82]	Case report	Low–mod	Autopsy confirmation	Low
Karatela 2025 [83]	Case report	Low–mod	Concordant CSF PCR	Low
Tran 2025 [84]	Case report	Low	Tissue confirmation	Low
Bristol 2025 [85]	Case report	Low–mod	Tissue confirmation	Low
Valencia Uribe 2025 [86]	Case report	Moderate	Delayed paired confirmation	High
Nelson 2025 [87]	Case report	Low–mod	Multilayer corroboration	Low
El Atrache 2025 [88]	Case report	Low	Tissue confirmation of miss	Low
Phillips 2025 [89]	Case report	Low–mod	Concordant CSF mNGS	Low
Chatterjee 2025 [90]	Case report	Moderate	No CNS tissue confirmation	High
Cohodes 2025 [91]	Case report	Moderate	CNS involvement not CSF-defined	High
Ladines-Lim 2025 [92]	Case report	Low–mod	Culture confirmation	Low
Alhaj 2025 [93]	Case report	High	Infection unconfirmed	High
Jiang 2026 [94]	Case report	High	Low reads; causality uncertain	High
Peng 2026 [95]	Case report	Moderate	No histology	High
Koirala 2026 [96]	Case report	Moderate	No culture confirmation	High
Wong 2026 [97]	Case report	Low	Tissue confirmation	Low
Kaira 2026 [98]	Case report	Low–mod	Concordant CSF PCR	Low
Wilson 2026 [99]	Case report	High	Abstract-level	Unclear

Abbreviations: CDC, United States Centers for Disease Control and Prevention; ciHHV-6, chromosomally integrated HHV-6; CNS, central nervous system; CSF, cerebrospinal fluid; FUO, fever of unknown origin; mNGS, metagenomic next-generation sequencing; Mod, moderate; NGS, next-generation sequencing; PCR, polymerase chain reaction; Retrosp., retrospective; WGA, whole-genome amplification. Concern levels are qualitative summaries (QUADAS-2 domains for cohorts; case-level domains for series and case reports) and are not validated numerical scores. § Three-category judgement, mapped from the descriptive label: low and low–moderate to low; moderate, moderate–high, and high to high; unclear for abstract-level extractions in which a domain could not be judged. This is the QUADAS-2 result; the descriptive label is retained only for the reason assigned. Neither instrument contains a sponsorship domain.

## Data Availability

No new data were created or analyzed in this study.

## Supplementary Materials

The following supporting information can be downloaded at: https://www.mdpi.com/article/10.3390/diagnostics16203375/s1, Table S1: PRISMA Checklist.

## Abbreviations

The following abbreviations are used in this manuscript:

cfDNA	cell-free DNA
CNS	central nervous system
CSF	cerebrospinal fluid
HHV-6	human herpesvirus 6
mcfDNA	microbial cell-free DNA
mNGS	metagenomic next-generation sequencing
NR	not reported
PCR	polymerase chain reaction
PICOS	population, index test, comparator, outcomes, study design
PRISMA	Preferred Reporting Items for Systematic Reviews and Meta-Analyses
QUADAS-2	Quality Assessment of Diagnostic Accuracy Studies 2
TAT	turnaround time

## Appendix A

Table A1 reproduces the PubMed/MEDLINE search strings with the number of records each retrieved; Table A2 lists every report excluded after full-text assessment, and every report that could not be retrieved, with its primary reason.

**Table A1:** Search strings.

String	Search Expression	Records
S1	(plasma OR blood OR serum)[tiab] AND (“metagenomic next-generation sequencing” OR mNGS OR “cell-free DNA” OR cfDNA OR Karius)[tiab] AND (meningitis OR encephalitis OR meningoencephalitis OR “brain abscess” OR “intracranial abscess” OR ventriculitis)[tiab]	160
S2	(plasma OR blood OR serum)[tiab] AND (“cell-free DNA” OR cfDNA OR mcfDNA OR Karius OR “metagenomic next-generation sequencing” OR mNGS OR “metagenomic sequencing”)[tiab] AND (“central nervous system” OR CNS OR neuroinvasive OR “intracranial infection” OR “cerebral abscess” OR rhombencephalitis OR “mycotic aneurysm”)[tiab]	240
S3	Karius[tiab] OR “microbial cell-free DNA”[tiab] OR mcfDNA[tiab] OR “plasma cell-free DNA sequencing”[tiab] OR “plasma metagenomic”[tiab]	309
S4	(“cell-free DNA” OR cfDNA OR “next-generation sequencing” OR metagenomic)[tiab] AND (plasma OR serum)[tiab] AND (“aseptic meningitis” OR “tuberculous meningitis” OR “cryptococcal meningitis” OR “fungal meningitis” OR “amoebic encephalitis” OR “amebic encephalitis” OR “viral encephalitis” OR “bacterial meningitis”)[tiab]	26
S5	(“cell-free DNA” OR “cell free DNA” OR “cell-free plasma” OR mcfDNA OR Karius)[tiab] AND (metagenomic OR “next-generation sequencing” OR “next generation sequencing”)[tiab] AND (infection OR infections OR infectious OR pathogen OR pathogens)[tiab] AND (plasma OR blood)[tiab]	192
S6	(“next-generation sequencing” OR “next generation sequencing” OR metagenomic OR “cell-free”)[tiab] AND (plasma OR blood OR serum)[tiab] AND (meningitis OR encephalitis OR meningoencephalitis OR “brain abscess” OR “brain abscesses” OR ventriculitis OR “intracranial infection” OR cerebral)[tiab] AND (“case report”[tiab] OR “case series”[tiab] OR “Case Reports”[Publication Type])	217
S7	(“cell-free DNA” OR “cell free DNA” OR mcfDNA)[tiab] AND (metagenomic OR metagenomics)[tiab] AND (plasma OR blood OR serum)[tiab]	122
Total	All strings; 470 duplicates removed by PMID	1266 → 796 unique

Abbreviations: cfDNA, cell-free DNA; CNS, central nervous system; Karius, Karius plasma microbial cell-free DNA test; mcfDNA, microbial cell-free DNA; mNGS, metagenomic next-generation sequencing; PMID, PubMed identifier. The arrow (→) indicates the number of unique records remaining after removal of duplicates.

**Table A2:** Excluded and unretrieved reports.

Report	PMID	Primary Reason	Stage
Chen 2022	36250052	Blood fraction not specified	Excluded after assessment
Chen 2023	36607873	Blood fraction not specified	Excluded after assessment
Cui 2024	38166638	Blood fraction not specified	Excluded after assessment
Dai 2026b	41797768	Blood fraction not specified	Excluded after assessment
Du 2022	35874573	Blood fraction not specified	Excluded after assessment
Gu 2022	35665349	Blood fraction not specified	Excluded after assessment
Han 2025	40010603	Blood fraction not specified	Excluded after assessment
Huang 2025	40911167	Blood fraction not specified	Excluded after assessment
Ji 2024	39278332	Blood fraction not specified	Excluded after assessment
Kufner 2019	31470675	Blood fraction not specified	Excluded after assessment
Li 2021	34856692	Blood fraction not specified	Excluded after assessment
Li 2023	37904422	Blood fraction not specified	Excluded after assessment
Li 2026	42011182	Blood fraction not specified	Excluded after assessment
Liang 2026	41759636	Blood fraction not specified	Excluded after assessment
Liu 2019	31654372	Blood fraction not specified	Excluded after assessment
Liu 2021	34631726	Blood fraction not specified	Excluded after assessment
Liu 2022	35459229	Blood fraction not specified	Excluded after assessment
Liu 2024	39267516	Blood fraction not specified	Excluded after assessment
Qu 2023	38223708	Blood fraction not specified	Excluded after assessment
Sun 2023	38008898	Blood fraction not specified	Excluded after assessment
Wang 2020	31898060	Blood fraction not specified	Excluded after assessment
Wang 2026	42427959	Blood fraction not specified	Excluded after assessment
Wang 2026	41868024	Blood fraction not specified	Excluded after assessment
Wen 2021	34977080	Blood fraction not specified	Excluded after assessment
Wu 2025	40104149	Blood fraction not specified	Excluded after assessment
Xu 2022b	35663067	Blood fraction not specified	Excluded after assessment
Yan 2022	35096860	Blood fraction not specified	Excluded after assessment
Yang 2025	41357797	Blood fraction not specified	Excluded after assessment
Ye 2024	38728637	Blood fraction not specified	Excluded after assessment
Zeng 2021	34568372	Blood fraction not specified	Excluded after assessment
Zhang 2022	35646766	Blood fraction not specified	Excluded after assessment
Zhang 2026	42457325	Blood fraction not specified	Excluded after assessment
Zhao 2025	41365462	Blood fraction not specified	Excluded after assessment
Zhao 2026	42436393	Blood fraction not specified	Excluded after assessment
Chen 2023b	36889461	CNS plasma result not separable	Excluded after assessment
Dai 2026	41864933	CNS plasma result not separable	Excluded after assessment
Duan 2021	33435894	CNS plasma result not separable	Excluded after assessment
Francisco 2023 (listed as ‘Ashe plasma NGS antimicrobial management 2023’; ASHE is the journal)	36865703	CNS plasma result not separable	Excluded after assessment
Gracia 2024	38502901	CNS plasma result not separable	Excluded after assessment
Guo 2021	35070842	CNS plasma result not separable	Excluded after assessment
Heldman 2024	37405403	CNS plasma result not separable	Excluded after assessment
Lee 2020	32376666	CNS plasma result not separable	Excluded after assessment
Leon 2020	32139440	CNS plasma result not separable	Excluded after assessment
Meng 2022	36110128	CNS plasma result not separable	Excluded after assessment
Park 2023	37439686	CNS plasma result not separable	Excluded after assessment
Resman Rus 2025	39859368	CNS plasma result not separable	Excluded after assessment
Rossoff 2019	31375834	CNS plasma result not separable	Excluded after assessment
Shah 2024	39717490	CNS plasma result not separable	Excluded after assessment
Sun 2026	41481221	CNS plasma result not separable	Excluded after assessment
Wang 2021	34124149	CNS plasma result not separable	Excluded after assessment
Wang 2026b	41836379	CNS plasma result not separable	Excluded after assessment
Zhang 2024	38347444	CNS plasma result not separable	Excluded after assessment
Zhu 2023	37260394	CNS plasma result not separable	Excluded after assessment
Ba 2025	41043636	Full text not retrieved	Sought; not retrieved
Deas 2026	42586789	Full text not retrieved	Sought; not retrieved
Esse 2025	39978635	Full text not retrieved	Sought; not retrieved
Jang 2024	39146635	Full text not retrieved	Sought; not retrieved
Lee 2025	40720094	Full text not retrieved	Sought; not retrieved
Niles 2022	34845152	Full text not retrieved	Sought; not retrieved
Shen 2023	36724855	Full text not retrieved	Sought; not retrieved
Thompson 2024	38843606	Full text not retrieved	Sought; not retrieved
Weiss 2023	37467522	Full text not retrieved	Sought; not retrieved
Wu 2023	36908212	Full text not retrieved	Sought; not retrieved
Xu 2022	36462587	Full text not retrieved	Sought; not retrieved
Yi 2020	33887622	Full text not retrieved	Sought; not retrieved
Yu 2021	33849818	Full text not retrieved	Sought; not retrieved
Adolph 2026	41949810	No CNS infection	Excluded after assessment
Allen 2025	39749435	No CNS infection	Excluded after assessment
Balks 2024	39143203	No CNS infection	Excluded after assessment
Banz 2026	42321936	No CNS infection	Excluded after assessment
Blauwkamp 2019	30742071	No CNS infection	Excluded after assessment
Brandon 2025	40692859	No CNS infection	Excluded after assessment
Centeno 2024	38415091	No CNS infection	Excluded after assessment
Chen 2024	38371300	No CNS infection	Excluded after assessment
Cheng 2022	35058359	No CNS infection	Excluded after assessment
Emeagui 2025	41523543	No CNS infection	Excluded after assessment
Feng 2023	37271194	No CNS infection	Excluded after assessment
Fung 2018	30581881	No CNS infection	Excluded after assessment
Fung 2024	39086465	No CNS infection	Excluded after assessment
Graf 2025	39749434	No CNS infection	Excluded after assessment
Gundelly 2025	40968586	No CNS infection	Excluded after assessment
Hogan 2021	31942944	No CNS infection	Excluded after assessment
Issa 2025	40688428	No CNS infection	Excluded after assessment
Jenkins 2025	40233994	No CNS infection	Excluded after assessment
Kalbitz 2024	38777941	No CNS infection	Excluded after assessment
Kaur 2026	41527828	No CNS infection	Excluded after assessment
Lyu 2026	42359352	No CNS infection	Excluded after assessment
Madut 2024	39091643	No CNS infection	Excluded after assessment
Neidhofer 2025	39949721	No CNS infection	Excluded after assessment
Ogunsiakan 2022	35368989	No CNS infection	Excluded after assessment
Petri 2024	38989532	No CNS infection	Excluded after assessment
Roy 2020	33194865	No CNS infection	Excluded after assessment
Saad 2025	41177684	No CNS infection	Excluded after assessment
Saleem 2026	42404677	No CNS infection	Excluded after assessment
Smith 2024	39477448	No CNS infection	Excluded after assessment
Vasselin 2026	42094994	No CNS infection	Excluded after assessment
Vinh Dong 2024	38167775	No CNS infection	Excluded after assessment
Vissichelli 2023	36632004	No CNS infection	Excluded after assessment
Wang 2022b	36547776	No CNS infection	Excluded after assessment
Wang 2024	38725449	No CNS infection	Excluded after assessment
Watson 2025	41160772	No CNS infection	Excluded after assessment
Wilke 2021	34112116	No CNS infection	Excluded after assessment
Williams 2024	38404751	No CNS infection	Excluded after assessment
Yin 2025	39008608	No CNS infection	Excluded after assessment
Zhang 2025	40415957	No CNS infection	Excluded after assessment
Olthoff 2022 (IDWeek abstract)	—	Not a primary report	Excluded after assessment
Ryder 2026	42038636	Not a primary report	Excluded after assessment
Cao 2026	42548546	Not plasma/serum metagenomics	Excluded after assessment
Chen 2024b	39323466	Not plasma/serum metagenomics	Excluded after assessment
Chen 2025	40861207	Not plasma/serum metagenomics	Excluded after assessment
Cong 2025	41112953	Not plasma/serum metagenomics	Excluded after assessment
DU 2026	42130363	Not plasma/serum metagenomics	Excluded after assessment
Gudenkauf 2025	42111477	Not plasma/serum metagenomics	Excluded after assessment
Han 2024	38433784	Not plasma/serum metagenomics	Excluded after assessment
Horiba 2022	36299531	Not plasma/serum metagenomics	Excluded after assessment
Hou 2023	37674578	Not plasma/serum metagenomics	Excluded after assessment
Howard-Jones 2025	40550235	Not plasma/serum metagenomics	Excluded after assessment
Huang 2021	33717113	Not plasma/serum metagenomics	Excluded after assessment
Li 2022	35845529	Not plasma/serum metagenomics	Excluded after assessment
Li 2022b	35529871	Not plasma/serum metagenomics	Excluded after assessment
Li 2025	40678340	Not plasma/serum metagenomics	Excluded after assessment
Li 2026b	42540632	Not plasma/serum metagenomics	Excluded after assessment
Liu 2024	39578877	Not plasma/serum metagenomics	Excluded after assessment
Liu 2025	41310694	Not plasma/serum metagenomics	Excluded after assessment
Liu 2026	42473611	Not plasma/serum metagenomics	Excluded after assessment
Ma 2021	33693022	Not plasma/serum metagenomics	Excluded after assessment
Qu 2022	35509305	Not plasma/serum metagenomics	Excluded after assessment
Rubbab 2026	42342687	Not plasma/serum metagenomics	Excluded after assessment
Tan 2024	38898852	Not plasma/serum metagenomics	Excluded after assessment
Wang 2023	37024789	Not plasma/serum metagenomics	Excluded after assessment
Yang 2025	40855467	Not plasma/serum metagenomics	Excluded after assessment
Zhao 2021	33866842	Not plasma/serum metagenomics	Excluded after assessment
Zhao 2025	40060957	Not plasma/serum metagenomics	Excluded after assessment
Zou 2024	38563313	Not plasma/serum metagenomics	Excluded after assessment

Abbreviations: CNS, central nervous system; NGS, next-generation sequencing; PMID, PubMed identifier. The final column sums to 121 reports excluded after assessment and 13 sought but not retrieved, giving the 134 rows listed. It records the stage at which each report left the review rather than the arm in which it was identified, because every listed record carries a PubMed identifier and the 2 other-methods exclusions are not separable here.
